# The Energetic Viability of Δ^1^-Piperideine Dimerization in Lysine-derived Alkaloid Biosynthesis

**DOI:** 10.3390/metabo8030048

**Published:** 2018-08-31

**Authors:** Hajime Sato, Masanobu Uchiyama, Kazuki Saito, Mami Yamazaki

**Affiliations:** 1 Graduate School of Pharmaceutical Sciences, Chiba University, 1-8-1 Inohana, Chuo-ku, Chiba 260-8675, Japan; hajime.sato@chiba-u.jp (H.S.); ksaito@faculty.chiba-u.jp (K.S.); 2 Cluster of Pioneering Research (CPR), Advanced Elements Chemistry Laboratory, RIKEN, 2-1 Hirosawa, Wako, Saitama 351-0198, Japan; uchiyama@mol.f.u-tokyo.ac.jp; 3 Graduate School of Pharmaceutical Sciences, The University of Tokyo, 7-3-1 Hongo, Bunkyo-ku, Tokyo 113-0033, Japan; 4 RIKEN Center for Sustainable Resource Science (Yokohama campus), 1-7-22 Suehiro-cho, Tsurumi-ku, Yokohama 230-0045, Japan

**Keywords:** alkaloid, quinolizidine, lysine, plant, piperideine, nitramidine, DFT calculation

## Abstract

Lys-derived alkaloids widely distributed in plant kingdom have received considerable attention and have been intensively studied; however, little is known about their biosynthetic mechanisms. In terms of the skeleton formation, for example, of quinolizidine alkaloid biosynthesis, only the very first two steps have been identified and the later steps remain unknown. In addition, there is no available information on the number of enzymes and reactions required for their skeletal construction. The involvement of the $\Delta^{1}$-piperideine dimerization has been proposed for some of the Lys-derived alkaloid biosyntheses, but no enzymes for this dimerization reaction have been reported to date; moreover, it is not clear whether this dimerization reaction proceeds spontaneously or enzymatically. In this study, the energetic viability of the $\Delta^{1}$-piperideine dimerizations under neutral and acidic conditions was assessed using the density functional theory computations. In addition, a similar type of reaction in the dipiperidine indole alkaloid, nitramidine, biosynthesis was also investigated. Our findings will be useful to narrow down the candidate genes involved in the Lys-derived alkaloid biosynthesis.

## 1. Introduction

In comparison to the biosynthetic studies in microorganisms, the elucidation of the whole biosynthetic genes responsible for the production of a secondary metabolite in plant is still challenging, even with the recent breakthrough technologies such as omics studies [1,2]. Furthermore, it is still difficult to narrow down candidate genes involved in a biosynthetic pathway, for instance plant alkaloid biosynthesis, as the number of reactions and intermediates involved in many of them are still unclear. Recently, computational chemistry has been used for deep understanding of the reaction mechanisms or searching for the pathways in many types of natural product biosynthesis, such as flavonoid biosynthesis [3], terpene cyclization [4], Diels–Alder reaction [5], P450-catalyzing reaction [6] and alkaloid formation [7]. For detailed elucidation of plant alkaloid biosynthetic pathways, besides traditional experimental approach, it would be quite helpful if the number of reactions and intermediates could be clarified by computational chemistry.

Quinolizidine alkaloids (QAs) [8], a subclass of Lys-derived plant alkaloids, widely distributed in *Leguminosae* [9,10], exhibit various biological activities such as cytotoxicity, anti-arrhythmic, oxytotic, hypoglycemic and anti-pyrogenic activity, thereby could be the potential medicinal seeds or pest control of plants. Accordingly, the elucidation of QA biosynthesis is also very important. Various type of QAs, constructed with a C5-building block cadaverine, have been reported, such as matrine, lupanine, multiflorine and lupinine, and have attracted many scientists due to their biosynthetic complexity and the inspirational value to contemporary (potentially) synthetic chemists.

Although much effort has been made to clarify how QAs are synthesized in plant, the reaction mechanisms behind QA biosynthesis are not fully understood. In the 1970s, a considerable number of labeling experiments were carried out by Spenser et al. [11,12,13,14,15,16] and insightful results were obtained for the first half of the quinolizidine skeleton formation, but the latter part is still unclear. We have been working on QA biosynthesis, and have reported several enzymes involved in their biosynthesis, such as Lysine decarboxylase(LDC) [17], acyltransferase [18,19] and *O*-tigloyltransferase [20,21]. In addition, a copper amine oxidase (CAO) from *Lupinus*, *La*CAO, catalyzing the second step in QA biosynthesis, was recently reported by Yang et al [22]. Although the very first part of skeletal construction and the following modification steps were revealed, the key reaction steps in QA skeleton formation, such as tri- and tetracyclic formation, oxidation, etc., have not yet been elucidated.

**Scheme 1:**
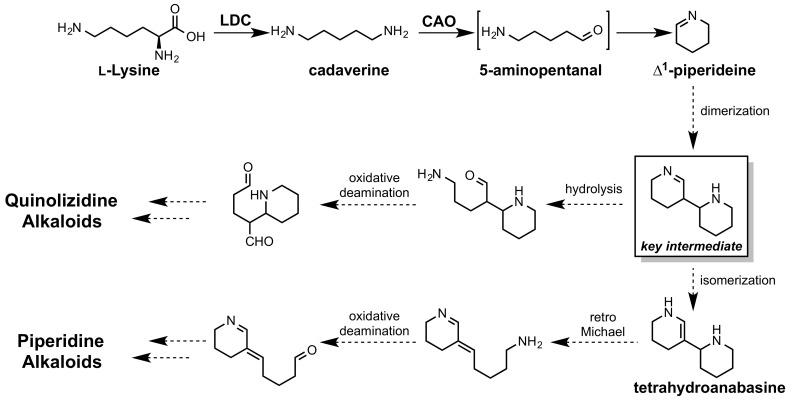
The plausible biosynthetic pathway for Lys-derived alkaloid. Both quinolizidine alkaloids and piperidine alkaloids are synthesized from L-Lys. Piperideine dimer is the common intermediate for both biosynthetic pathways.

The mechanism widely accepted for the formation of QA, shown in Scheme 1, proposed the involvement of $\Delta^{1}$-piperideine dimerization reaction, which has not yet been experimentally validated to date. Whether this Mannich-aldol type dimerization reaction proceeds under the biomimetic conditions is also unclear. Wanner and Koomen [23] mentioned “this imine (meaning $\Delta^{1}$-piperideine) is extremely reactive and dimerizes spontaneously in water at neutral pH to form tetrahydroanabasine”. In contrast, they also mentioned “$\Delta^{1}$-piperideine and tetrahydroanabasine are too unstable to be obtained as such from plant material” [23]. In fact, they isolated tetrahydroanabasine as a HBr salt, which makes it difficult to exclude the possibility of acid-catalyzed dimerization.

In this study, we carried out DFT calculation to clarify the feasibility of $\Delta^{1}$-piperideine dimerization reaction, which can provide a new insight for the other Lys-derived alkaloids analysis. We also demonstrate a similar intramolecular dimerization-type reaction in the biosynthesis of a dipiperidine indole alkaloid, nitramidine, which is also thought to be synthesized from cadaverine as well as QAs (Scheme 1).

## 2. Result

### 2.1. Benchmark Calculations

**Figure 1:**
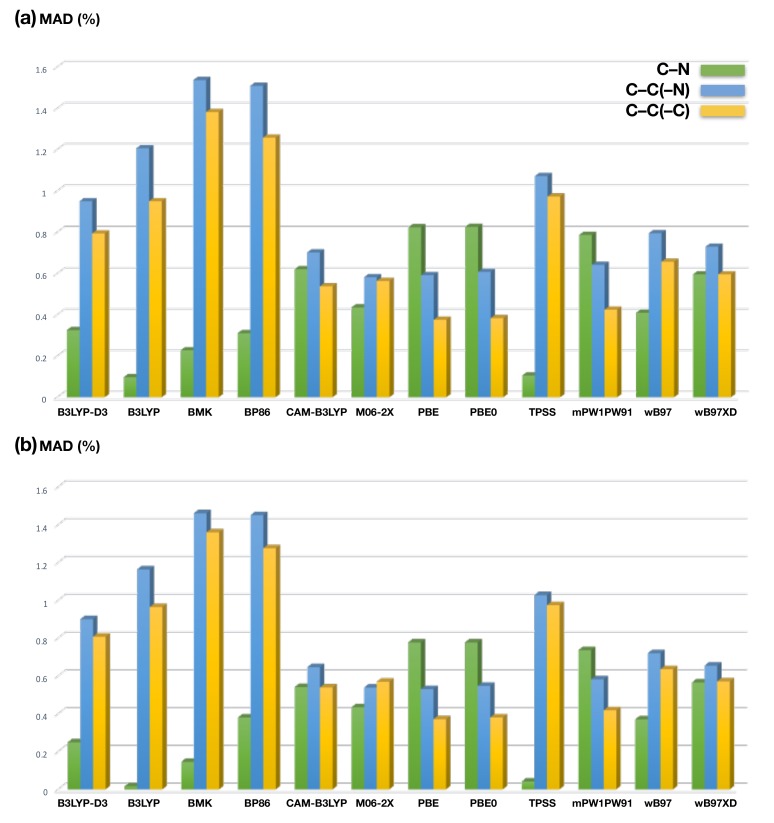
Benchmark result for isotripiperideine geometry optimization, where green bar represents C–N bond distance, blue bar represents C–C bond distance adjacent to nitrogen atom and yellow bar represents C–C bond distance adjacent to carbon atom: (**a**) benchmark calculation with 6-31G(d) Basis set; and (**b**) benchmark calculation with 6-31G+(d,p) Basis set.

To ascertain which density functional was likely to describe the energetics of Lys-derived alkaloid biosynthesis, we optimized the isotripiperideine ($\Delta^{1}$-piperideine trimer) structure and compared the bond lengths of C–C(–C), C–C(–N) and C–N. We used the X-ray crystal structure of isotripiperideine [24] as a reference. We tested 12 different functionals (B3LYP-D3, B3LYP [25], BMK [26], BP86, CAM-B3LYP [27], M06-2X [28], PBE [29], PBE0 [30], TPSS [31], mPW1PW91 [32], wB97 [33] and wB97XD [34], where “D3” indicates the explicit inclusion of Grimme’s D3 atom-pairwise dispersion correction [35]) with two different level of basis set: 6-31G(d) and 6-31+G(d,p). The overall performance of the tested functionals can be best gauged by inspecting their mean absolute deviations (MADs in Figure 1). All MADs are shown in percentage relative to the bond length in crystal structure.

BMK and BP86 are clearly associated with the poorest overall performance (MADs for C–C bonds are over 1.2%). B3LYP also showed very poor performance, and was not slightly improved by adding the dispersion correction (B3LYP-D3). For the C–C(–C) bond or the C–C(–N) bond, PBE, PBE0 and mPW1PW91 showed relatively better performance, however they showed poor result for the C–N bond length. Considering of the average MAD for three different bond types, M06-2X showed the best performance with both 6-31G(d) and 6-31+G(d,p). Thus, we believe that M06-2X/6-31+G(d,p) should prove to be a reliable producer for the investigation of Lys-derived alkaloid biosynthesis. The remaining energy comparisons presented in this work are made, therefore, using this level of theory.

### 2.2. $\Delta^{1}$-Piperideine Dimerization

**Scheme 2:**
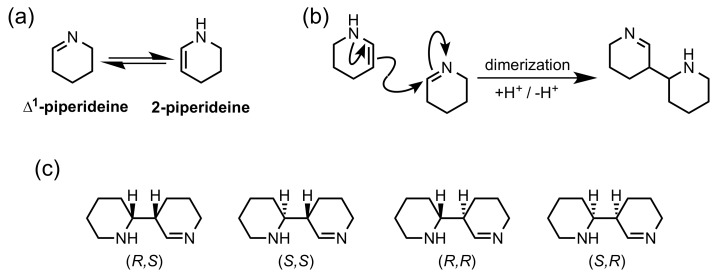
The plausible biosynthetic pathway for the $\Delta^{1}$-piperideine dimerization: (**a**) the interconversion between $\Delta^{1}$-piperideine and 2-piperideine; (**b**) the scheme of $\Delta^{1}$-piperideine dimerization reaction; and (**c**) four possible isomers of piperideine dimers.

In QA biosynthesis, $\Delta^{1}$-piperideine is spontaneously formed from 5-aminopentanal, produced by CAO-catalyzed oxidation of cadaverine, which has been experimentally verified [22]. Then, $\Delta^{1}$-piperideine is thought to be interconverted to 2-piperideine under the biomimetic conditions [36] (Scheme 2a). Herein, we investigated the potential energy surface for the reaction between $\Delta^{1}$-piperideine and 2-piperideine, that is, dimerization reaction (Scheme 2b). To obtain the reasonable transition state structures, we first carried out the conformational search for the four possible isomers of piperideine dimers shown in Scheme 2c. More than 50 conformers were found for each isomer, and all conformers were optimized at the M06-2X/6-31+G(d,p) level of theory. Subsequently, 2–4 molecules of water were located around each conformer to donate/accept protons, and then the potential energy surfaces for the dimerization reaction were scanned.

**Figure 2:**
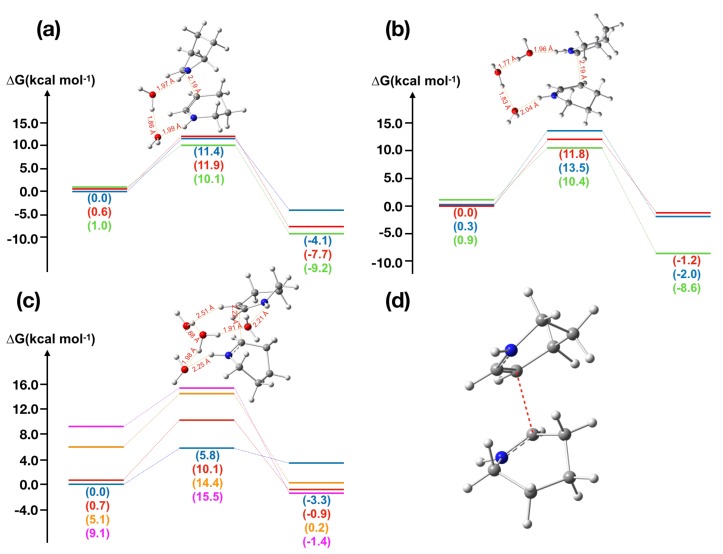
Computed energy profiles for $\Delta^{1}$-piperideine dimerization reaction under acidic conditions, where green and red lines are for (*R,S*)-piperideine dimers and blue, orange and purple lines are for (*S,R*)-piperideine dimers: (**a**) dimerization with two molecules of water; (**b**) dimerization with three molecules of water; (**c**) dimerization with four molecules of water; and (**d**) transition state structure of piperideine dimerization reaction under acidic conditions.

In the retro-conversion of (*R,R*)- or (*S,S*)-piperideine dimers to $\Delta^{1}$-piperideine, as the distance between two piperideine rings are elongated, the energies kept on increasing, indicating (*R,R*)- and (*S,S*)-piperideine dimer formation could spontaneously take place (Figures S1 and S2). In contrast, several transition state structures were found in (*R,S*)- and (*S,R*)-piperideine dimer formation, which require over 20 kcal mol${}^{-1}$ under neutral conditions and ca. 10 kcal mol${}^{-1}$ under acidic conditions (Figure 2a–c). As it is commonly accepted that a chemical reaction requiring lower than 20–25 kcal mol${}^{-1}$ can proceed under ambient conditions [37], (*R,S*)- and (*S,R*)-piperideine dimer formation might require enzymatic support or acidic conditions. However, we cannot rule out the possibility that chemical formation takes place very slowly in plant, since its activation energy, 21.7 kcal mol${}^{-1}$, is in a marginal range allowing spontaneous chemical reaction (Figure S3). Interestingly, the transition state structure for the conversion of (*R,S*)- and (*S,R*)-piperideine dimer forms a sandwich-type structure, where two piperideine rings are facing each other (Figure 2d).

### 2.3. Nitramidine Biosynthesis

**Scheme 3:**
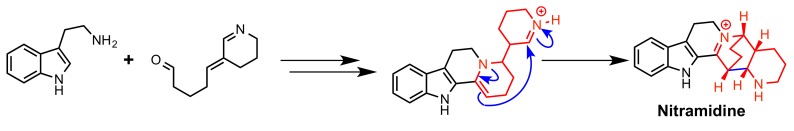
The plausible biosynthetic mechanisms for nitramidine.

The dipiperidine indole alkaloid nitramidine has received the attention of organic chemists due to its complex, stereodense, polycyclic and nitrogen rich structures. Although several biomimetic syntheses were reported [38], the details of their biosynthesis are still unknown. Nitramidine isolated from *Nitraria schoberi* [39] is thought to be biosynthesized from the pentacyclic intermediate (Scheme 3) [40]; the reaction can be viewed as a variation of the piperideine dimerization. We therefore examined the feasibility of this piperideine dimerization-type reaction.

**Figure 3:**
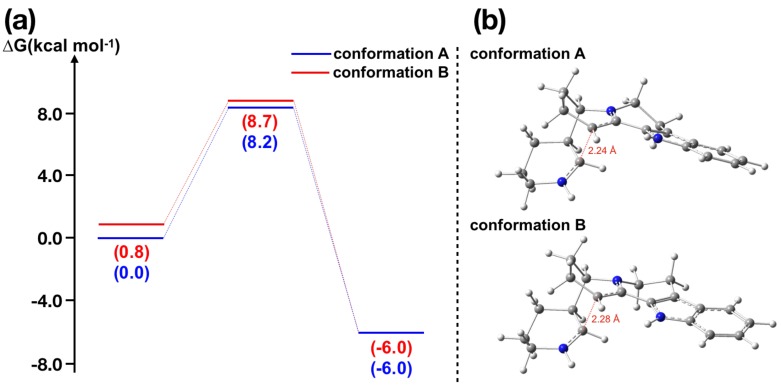
Calculation results of piperideine dimerization-type reaction in nitramidine biosynthesis: (**a**) energy diagram for the nitramidine dimerization-like reaction; and (**b**) transition state structures of nitramidine dimerization-like reaction for two conformers.

Only two conformers were found for nitramidine due to its rigid structure, and both were subjected to the transition state search. No transition state structure was obtained under neutral conditions; as both rings come closer, the energy kept increasing. As with the intermolecular piperideine dimerization, transition state structures were found under acidic condition, the activation energy is ca. 8.5 kcal mol${}^{-1}$ (Figure 3a). Interestingly, the transition state structures of both nitramidine conformers also form the sandwich-type structure (Figure 3b).

## 3. Discussion

Based on our calculation, the formation of (*R,R*)- and (*S,S*)-piperideine dimer could take place without an enzymatic support, whereas the formation of (*S,R*)- and (*R,S*)-piperideine dimer requires over 20 kcal mol${}^{-1}$ energies under neutral conditions and ca. 10 kcal mol${}^{-1}$ energies under acidic conditions, respectively. As shown in Figure 2d, (*S,R*)- and (*R,S*)-piperideine dimerization reactions proceed through a sandwich-type transition state structure, indicating that the steric repulsion between the piperideine rings might increase the energy barrier. According to the previous paper [22], *La*CAO localizes in peroxisome in plant, which does not provide the strong acidic condition, thereby (*S,R*)- and (*R,S*)-piperideine dimers are not likely produced spontaneously in plant.

The tautomerization between $\Delta^{1}$-piperideine and 2-piperideine is also controversial issue. It can be viewed as a simple imine-enamine tautomerization; however, in tryptophan biosynthesis, imine-enamine tautomerization is catalyzed by the enzyme *TrpF* [41]. Indeed, piperideine dimers were not detected by an in vitro assay of *La*CAO [22], indicating this tautomerization might be the rate limiting step of this dimerization. Considering together that the interconversion between $\Delta^{1}$-piperideine and 2-piperideine appears to be relatively slow, we can postulate that once 2-piperideine was formed in plant, then the dimerization reaction proceeds instantly.

**Scheme 4:**
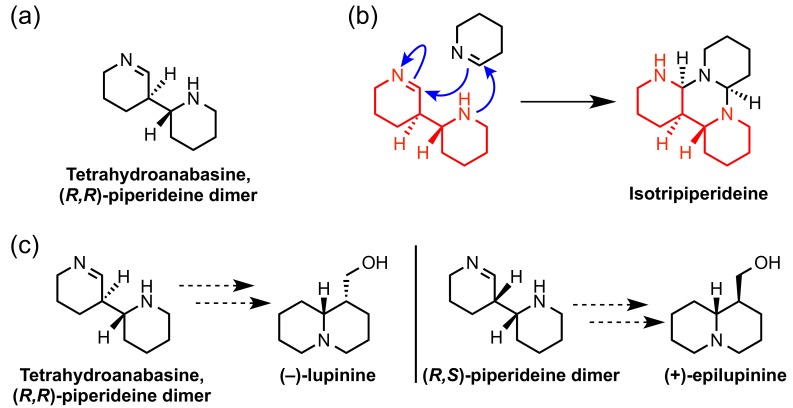
The stereochemistry of tetrahydroanabasine, isotripiperideine, (–)-lupinine and (+)-epilupinine: (**a**) the stereochemistry of tetrahydroanabasine; (**b**) the plausible mechanism of isopiperideine formation; and (**c**) the plausible mechanism of (–)-lupinine and (+)-epilupinine formation.

The stereochemistry of tetrahydroanabasine corresponds to the (*R,R*)-piperideine dimer (Scheme 4a) [15], and isotripiperideine appears to be synthesized from the (*R,R*)-piperideine dimer (Scheme 4b) [24], consistent with our calculation result. Notably, two representative C10 QAs, (–)-lupinine and (+)-epilupinine, also appear to be synthesized from (*R,R*)-piperideine and (*R,S*)-piperideine-dimer, respectively (Scheme 4c). The involvement of (*R,R*)-piperideine dimer as the biosynthetic intermediate of (–)-lupinine was previously reported by stable-isotope labeling experiments [15]. The detection of (+)-epilupinine suggests that (*R,S*)-piperideine dimer formation may take place in plant very slowly, as discussed in Section 2.2. However, (*S,S*)-piperideine dimer was not reported as a plant metabolite, indicating that an enzyme could mediates the formation of (*R,R*)-piperideine dimer or decompose (*S,S*)-piperideine dimer selectively. In thebaine biosynthesis, thebaine synthase (THS) catalyzes the conversion of (7*S*)-salutaridinol 7-*O*-acetate to thebaine, although this reaction spontaneously proceeds in aqueous conditions [42]. THS-catalyzed reaction can prevent the formation of hydroxylated byproducts, which is the benefit for regulating the bioactive product metabolism. This fact clearly shows that even if the reaction can proceed in vitro, the possibility of enzymatic assist cannot be completely ruled out. Accordingly, we can conclude that enzymatic barrier-lowering is not necessary for piperideine dimerization reaction under the biomimetic conditions in plant, but it is not surprising if this reaction could be catalyzed by an enzyme.

Next, we examined the intramolecular piperideine dimerization-type reaction in nitramidine biosynthesis to shed light on its biosynthesis, as our $\Delta^{1}$-piperideine dimerization calculation showed good agreement with the experimental data previously reported [36]. The stereochemistry of nitramidine corresponds to (*S,R*)-piperideine dimer, suggesting this reaction would require an enzymatic support. Upon searching the transition state structure, we could not find the transition state for the formation of nitramidine under neutral conditions, indicating the requirement of protonation to the $\Delta^{1}$-piperideine. The transition state structures were found only under acidic conditions, for which the activation energies are 8–9 kcal mol${}^{-1}$ (Figure 3a), consistent with the experimental result, in which HCl was used to form the nitramidine [38]. Interestingly, the activation energy was not decreased significantly in comparison to the intermolecular (*S,R*)-piperideine dimerization reaction, even though this is the intramolecular reaction, suggesting that the entropy is not the main factor of this reaction. Clearly, nitramidine is biosynthesized under the biomimetic condition in plant, thereby enzymatic barrier-lowering would be required and the possibility of spontaneous reaction is also ruled out.

## 4. Methods

All DFT calculations were performed with Gaussian 16 program [43]. Benchmark test for the QA-forming reaction, B3LYP [25], B3LYP-D3 [35], BMK [26], BP86, CAM-B3LYP [27], M06-2X [28], PBE [29], PBE0 [30], TPSS [31], mPW1PW91 [32], wB97 [33] and wB97XD [34] were used with the combination of 6-31G(d) or 6-31+G(d,p) basis set. Geometry optimizations for benchmark test were conducted in the gas phase, without any symmetry restrictions.

All stereo isomers of piperideine dimers were subjected to a conformational search by Gromacs [44]. From these conformational searches, greater than one hundred structures per isomer were identified and then fully optimized at the M06-2X/6-31+G(d,p) level of theory. Then, 2–4 molecules of water were added to all conformers and then subjected to the transition state search. At the same level of theory, calculations of vibrational frequency were carried out to confirm that each TS possesses only a single imaginary frequency but a local minimum has no imaginary frequency. Intrinsic reaction coordinate calculations [45,46,47,48,49] for all TSs were conducted. Solvation was evaluated by the self-consistent reaction field (SCRF) method using the polarizable continuum model (PCM) [50,51,52]. In this study, the Gibbs free energy was adopted as the basis for discussion.

## 5. Conclusions

**Scheme 5:**
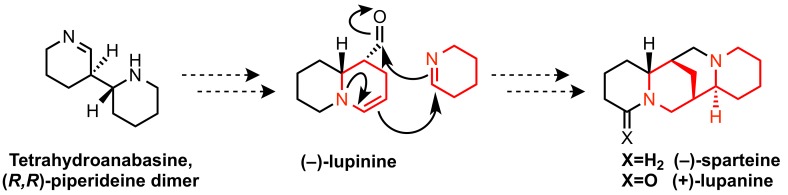
The plausible biosynthetic mechanisms for (–)-sparteine and (+)-lupanine.

Based on our benchmark test, M06-2X is the most suitable functional for the geometry optimization of Lys-derived alkaloid biosynthesis. The energetic viabilities of $\Delta^{1}$-piperideine dimerization reaction were assessed under both acidic and neutral conditions using DFT calculation. As a result, this dimerization reaction can spontaneously proceed when the reaction gives (*S,S*)- or (*R,R*)-piperideine dimers. The formation of (*S,R*)- and (*R,S*)-piperideine dimer require over 20 kcal mol${}^{-1}$ under neutral conditions and ca. 10 kcal mol${}^{-1}$ under acidic conditions, respectively. Therefore, we conclude that (*S,S*)- or (*R,R*)-piperideine dimers are spontaneously formed immediately after the conversion of $\Delta^{1}$-piperideine to 2-piperideine. However, we cannot rule out the possibility of enzymatic reaction, because nature uses an enzyme for the reaction that can spontaneously proceeds in aqueous conditions [42]. In addition, the detection of (+)-epilupinine suggests that (*R,S*)-piperideine dimer formation may take place very slowly in plant, as the activation energy is just the threshold value for the spontaneous reaction. However, C15 QA, such as (+)-lupanine and (–)-sparteine, are thought to be synthesized from (–)-lupinine-type intermediate (Scheme 5), indicating that (–)-lupinine is in the main stream in QA biosynthesis, whereas (+)-epilupinine could be the background reaction product or byproduct that would not undergo further enzymatic modifications in downstream.

Our detailed investigation on the dimerization mechanism of piperideine could further extend to examine the nitramidine biosynthesis. The stereochemistry of this reaction corresponds to (*S,R*)-piperideine dimer, therefore, the dimerization-type reaction in nitramidine biosynthesis would require an enzyme. Based on our calculation, intramolecular type dimerization in nitramidine biosynthesis requires acidic conditions or enzymatic support; this conclusion is consistent with the previous experimental result [38].

Overall, we show a computational basis on piperideine dimerization reaction, for which the activation energy is dependent on the stereochemistry of intermediates and products. This type of dimerization can be seen in various kinds of Lys-derived alkaloids biosynthesis, such as (+)-lupanine, (–)-sparteine, etc. (Scheme 5), as they are produced from the C5-building block cadaverine. The stereochemistry of the intramolecular dimerization reaction in (+)-lupanine and (–)-sparteine corresponds to (*R,S*)-piperideine dimer, suggesting that enzymatic-barrier lowering would not be required for this reaction. Our findings will be helpful for narrowing down the candidate genes if the exact stereochemistry of the products is available.

## Supplementary Materials

The following are available online at http://www.mdpi.com/2218-1989/8/3/48/s1. Figure S1: Computed energy profile for (S,S)-piperideine dimer formation under neutral conditions, Figure S2: Computed energy profile for (R,R)-piperideine dimer formation under neutral conditions, Figure S3: Computed energy profiles for Δ^1^-piperideine dimerization reaction under neutral conditions.

## Abbreviations

QA	quinolizidine alkaloid
LDC	lysine decarboxylase
THS	thebaine synthase
CAO	copper amine oxidase
DFT	density functional theory
