# Supplementary material for: The Energetic Viability of Δ1-Piperideine Dimerization in Lysine-derived Alkaloid Biosynthesis
Source: Metabolites. 2018 Aug 31;8(3):48. doi: 10.3390/metabo8030048 (PMC6161264; doi:10.3390/metabo8030048)
Supplement: Supplementary file 1 [file metabolites-08-00048-s001.pdf]

Supporting Information

The Energetic Viability of Δ[1]-Piperideine Dimerization in Lysine-derived Alkaloid Biosynthesis

Hajime Sato,[1,2] Masanobu Uchiyama,[2,3] Kazuki Saito,[1,4] and Mami Yamazaki[1,*]

1 Graduate School of Pharmaceutical Sciences, Chiba University, 1-8-1, Inohana, Chuo-ku, Chiba 260-8675, Japan

2 Cluster of Pioneering Research (CPR), Advanced Elements Chemistry Laboratory, 2-1 Hirosawa, Wako-shi, Saitama 351-0198, Japan

3 Graduate School of Pharmaceutical Sciences, University of Tokyo, 7-3-1 Hongo, Bunkyo-ku, Tokyo-to 113-0033, Japan

4 RIKEN Center for Sustainable Resource Science (Yokohama campus), 1-7-22 Suehiro-cho, Tsurumi-ku, Yokohama 230-0045, Japan.

*Email: mamiy@faculty.chiba-u.jp

Table of Contents

1.	General Methods	S-2
2.	Cartesian Coordinates	S-3
3.	References	S-54

1. General Methods

All DFT calculations were performed with Gaussian 16 program[1]. Benchmark test for the QA-forming reaction, B3LYP[2], B3LYP-D3[3], BMK[4], BP86, CAM-B3LYP[5], M06-2X[6], PBE[7], PBE0[8], TPSS[9], mPW1PW91[10], wB97[11] and wB97XD[12] were used with the combination of 6-31G(d) or 6-31+G(d,p) basis set. Geometry optimizations for benchmark test were conducted in the gas phase, without any symmetry restrictions.

All stereo isomers of piperideine dimers were subjected to a conformational search by Gromacs[13]. From these conformational searches, greater than one hundred structures per isomer were identified and then fully optimized at the M06-2X/6-31+G(d,p) level of theory. Then, two to four molecules of water were added to all conformers and then subjected to the transition state search. At the same level of theory, calculations of vibrational frequency were carried out to confirm that each TS possesses only a single imaginary frequency but a local minimum has no imaginary frequency. Intrinsic reaction coordinate calculations[14-18] for all TSs were conducted. Solvation was evaluated by the self-consistent reaction field (SCRF) method using the polarizable continuum model (PCM)[19-21]. In this study, the Gibbs free energy was adopted as the basis for discussion.

2. Cartesian Coordinates

2.1 Neutral conditions

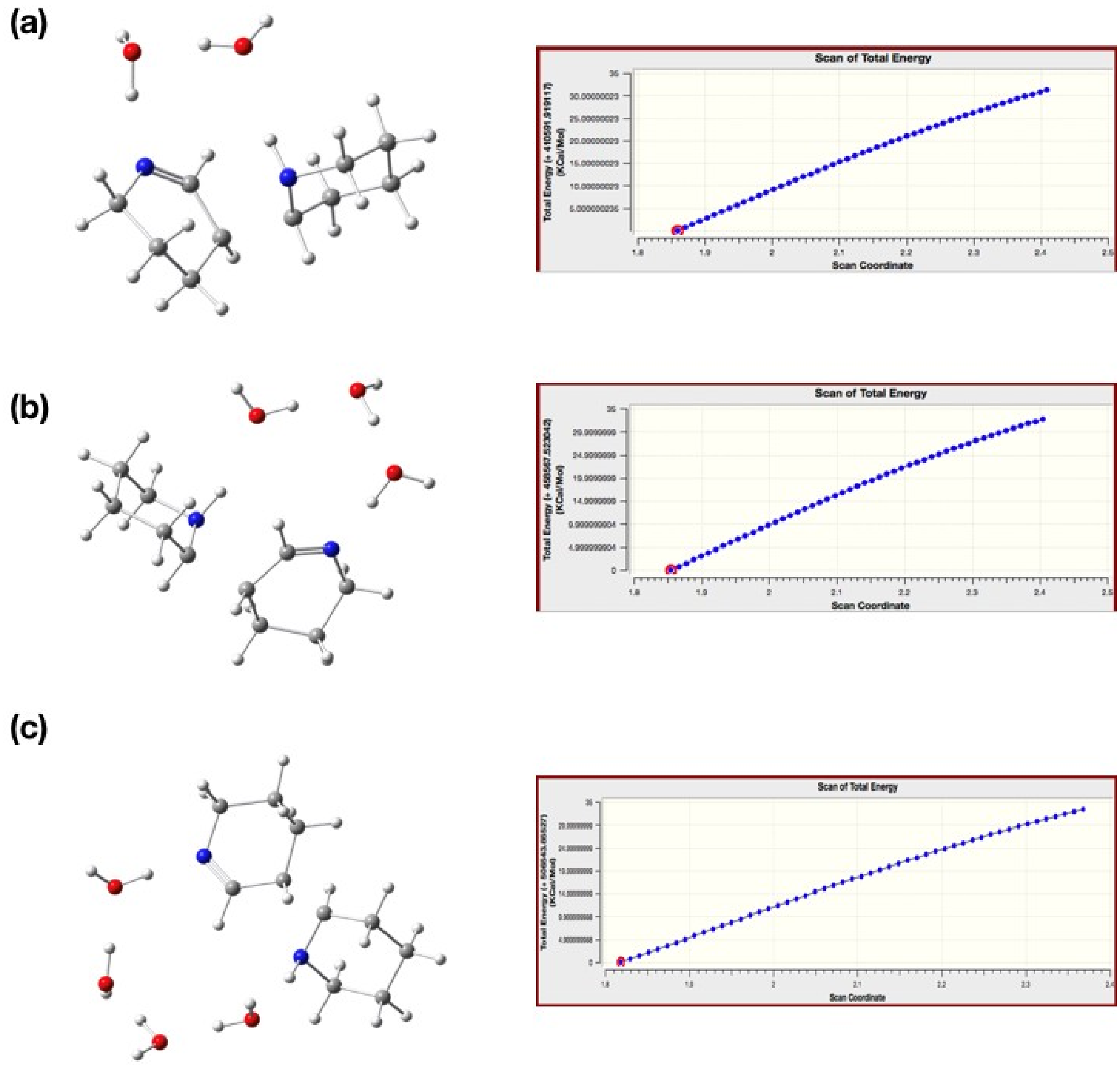

Figure S1. Computed energy profile for (*S,S*)-piperideine dimer formation under neutral conditions.
We have tested over 50 conformers with 2 to 4 molecules of water. As the C-C distance between two piperideine rings are elongated, the energies are increased.

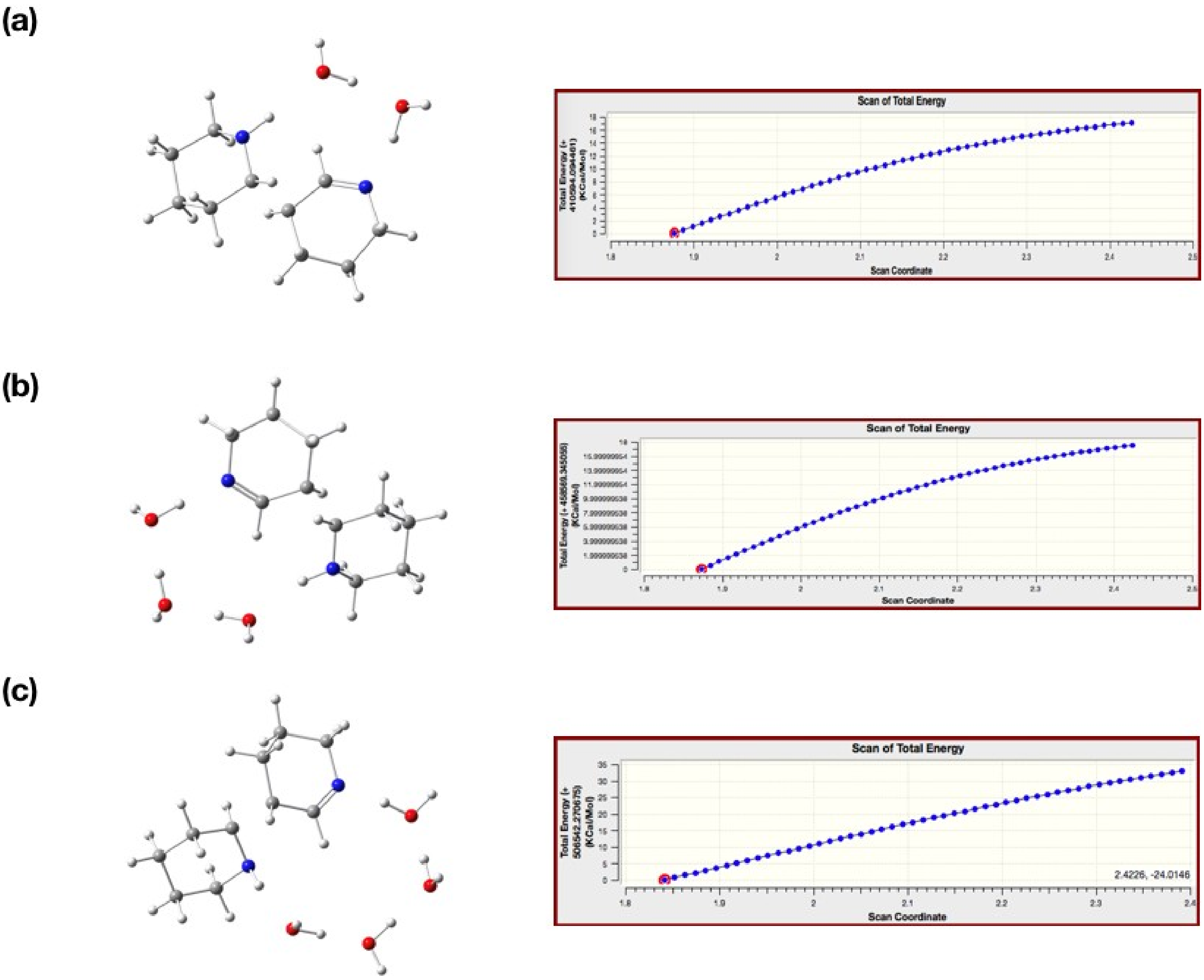

Figure S2. Computed energy profile for (*R,R*)-piperideine dimer formation under neutral conditions.
We have tested over 50 conformers with 2 to 4 molecules of water. As the C-C distance between two piperideine rings are elongated, the energies are increased.

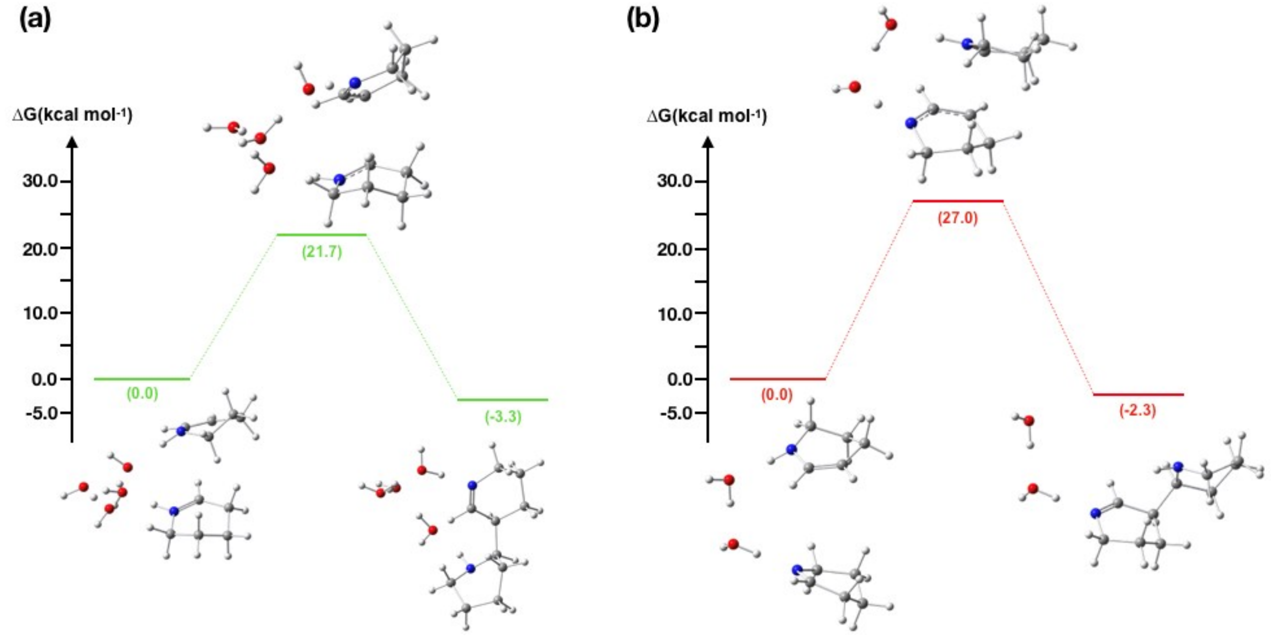

Figure S3. Computed energy profiles for Δ[1]-piperideine dimerization reaction under neutral conditions. (a): (*R,S*)-piperideine dimer formation, (b); (*S,R*)-piperideine dimer formation.

(R,S)_2H2O_neutral_F

Energy: -653.736852 hartree

Center Number	Atomic Number	Atomic Type	Coordinates (Angstroms) X	Y	Z
1	6	0	1.789091	1.122178	0.933235
2	6	0	1.186784	-0.359570	-0.937606
3	6	0	2.621700	-0.856824	-0.699770
4	6	0	3.586337	0.329275	-0.661702
5	6	0	3.274516	1.230572	0.550311
6	6	0	0.128216	-1.444646	-0.592498
7	6	0	0.015292	-1.864654	0.881509
8	6	0	-0.984406	-0.991285	1.640087
9	6	0	-2.340459	-1.039800	0.950175
10	7	0	0.992842	0.937527	-0.277415
11	7	0	-2.286367	-0.792984	-0.494721
12	6	0	-1.201460	-0.982630	-1.132896
13	1	0	1.449501	2.037435	1.427280
14	1	0	1.654080	0.304788	1.661221
15	1	0	2.692187	-1.406192	0.247251
16	1	0	2.886471	-1.562330	-1.493515

17	1	0	4.623125	-0.018472	-0.625222
18	1	0	3.473893	0.900549	-1.590424
19	1	0	3.889005	0.938753	1.409349
20	1	0	3.523611	2.269783	0.312027
21	1	0	1.088886	-0.186114	-2.021134
22	1	0	0.412198	-2.318744	-1.197587
23	1	0	-0.332461	-2.904065	0.921780
24	1	0	0.995229	-1.849314	1.366323
25	1	0	-1.089865	-1.338712	2.672536
26	1	0	-0.645580	0.049310	1.675154
27	1	0	-3.021564	-0.295295	1.374812
28	1	0	-2.815369	-2.019646	1.090597
29	1	0	-1.222597	-0.754226	-2.203846
30	1	0	0.011243	1.154079	-0.112451
31	8	0	-3.937638	1.305278	-0.925934
32	1	0	-4.055480	1.594013	-1.837717
33	1	0	-3.462400	0.433173	-0.959125
34	8	0	-1.723865	2.185998	0.557186
35	1	0	-1.633645	3.142561	0.630041
36	1	0	-2.527434	2.030948	0.022499

(R,S)_2H2O_neutral_R

Energy: -653.733133 hartree

Center Number	Atomic Number	Atomic Type	Coordinates (Angstroms) X	Y	Z
1	6	0	1.908667	-1.102013	0.942307
2	6	0	1.021810	-1.297683	-1.255992
3	6	0	0.041108	-2.378378	-0.897864
4	6	0	0.313348	-2.971634	0.483630
5	6	0	0.680911	-1.848351	1.451833
6	6	0	-1.875465	0.826553	-1.500215
7	6	0	-3.099002	0.242603	-0.833079
8	6	0	-2.820082	-0.068463	0.644243
9	6	0	-2.012821	1.055005	1.295923
10	7	0	1.853518	-0.732378	-0.476176
11	7	0	-0.748064	1.214727	0.595356
12	6	0	-0.818205	1.257652	-0.786367
13	1	0	2.816981	-1.702708	1.082214
14	1	0	2.057218	-0.172089	1.502027
15	1	0	-0.956485	-1.917254	-0.945484

16	1	0	0.061370	-3.141347	-1.683454
17	1	0	-0.560390	-3.527642	0.835909
18	1	0	1.145267	-3.683817	0.418624
19	1	0	-0.148955	-1.135530	1.524759
20	1	0	0.881555	-2.239253	2.454129
21	1	0	1.009605	-0.947285	-2.291631
22	1	0	-1.828605	0.869477	-2.583842
23	1	0	-3.950427	0.932165	-0.914272
24	1	0	-3.408582	-0.676068	-1.347373
25	1	0	-3.755473	-0.217568	1.191816
26	1	0	-2.244704	-0.998918	0.723077
27	1	0	-1.808093	0.825941	2.345982
28	1	0	-2.604568	1.985391	1.262366
29	1	0	0.079548	1.634670	-1.272249
30	1	0	2.555627	0.737904	-0.892776
31	8	0	1.613890	3.008358	1.002740
32	1	0	1.473469	3.933802	0.775431
33	1	0	-0.074128	1.859141	0.998197
34	8	0	2.832969	1.691709	-1.057767
35	1	0	3.795982	1.700130	-1.095664
36	1	0	2.109117	2.612134	0.251656

(R,S)_2H2O_neutral_TS

Energy: -653.690043 hartree

Center Number	Atomic Number	Atomic Type	Coordinates (Angstroms) X	Y	Z
1	6	0	1.425186	1.147229	1.397517
2	6	0	0.973213	0.637327	-0.993498
3	6	0	2.404314	0.193971	-1.241445
4	6	0	3.337293	0.665789	-0.123503
5	6	0	2.701766	0.333472	1.224112
6	6	0	-0.091059	-1.305478	-1.353797
7	6	0	0.374912	-2.343753	-0.361422
8	6	0	-0.188101	-2.057649	1.037475
9	6	0	-1.687675	-1.777338	0.985980
10	7	0	0.642935	1.197994	0.163491
11	7	0	-2.000134	-0.754969	-0.006206
12	6	0	-1.308659	-0.678932	-1.132285
13	1	0	1.679843	2.180002	1.663949
14	1	0	0.801216	0.749954	2.205050

15	1	0	2.467906	-0.893207	-1.325748
16	1	0	2.706102	0.598542	-2.212281
17	1	0	4.310590	0.180236	-0.229357
18	1	0	3.499222	1.747960	-0.196637
19	1	0	2.480250	-0.740913	1.263753
20	1	0	3.375475	0.556590	2.055264
21	1	0	0.431500	1.040584	-1.841632
22	1	0	0.273679	-1.354794	-2.375817
23	1	0	0.061338	-3.345306	-0.685647
24	1	0	1.471247	-2.379701	-0.314428
25	1	0	0.004834	-2.899858	1.708182
26	1	0	0.309958	-1.183119	1.464001
27	1	0	-2.052227	-1.432016	1.956684
28	1	0	-2.233654	-2.697579	0.736985
29	1	0	-1.697541	0.038875	-1.855058
30	1	0	-0.249494	1.720775	0.175698
31	8	0	-3.733226	1.149606	0.283849
32	1	0	-4.448555	1.190729	-0.360119
33	1	0	-2.810774	-0.054073	0.136609
34	8	0	-1.764992	2.543179	-0.251147
35	1	0	-1.883156	3.444753	0.065833
36	1	0	-2.658763	2.000285	-0.032694

(S,R)_4H2O_neutral_F

Energy: -806.524705 hartree

Center Number	Atomic Number	Atomic Type	Coordinates (Angstroms) X	Y	Z
1	6	0	-2.256817	-0.657367	0.421082
2	6	0	-0.183886	-1.962963	-0.171136
3	6	0	0.106145	-2.225859	1.332994
4	6	0	-1.136853	-2.034399	2.207174
5	6	0	-1.767796	-0.682063	1.880502
6	6	0	0.804720	-0.936316	-0.803800
7	6	0	2.207458	-1.542446	-0.928131
8	6	0	3.257246	-0.447516	-1.091987
9	6	0	3.197485	0.489880	0.108107
10	7	0	-1.605544	-1.699112	-0.382024
11	7	0	1.850890	1.005101	0.372802
12	6	0	0.831536	0.372305	-0.059331
13	1	0	-2.130709	0.350854	-0.005487

14	1	0	-3.332476	-0.866854	0.396194
15	1	0	0.514268	-3.232064	1.466373
16	1	0	0.879060	-1.530383	1.688555
17	1	0	-1.859202	-2.837773	2.025971
18	1	0	-0.853959	-2.080816	3.263551
19	1	0	-2.600940	-0.455079	2.552337
20	1	0	-1.010139	0.095977	2.048185
21	1	0	0.017631	-2.889375	-0.724245
22	1	0	0.411137	-0.706735	-1.806616
23	1	0	2.436918	-2.118288	-0.021299
24	1	0	2.228333	-2.246513	-1.765841
25	1	0	4.261785	-0.871858	-1.178460
26	1	0	3.061362	0.123891	-2.008188
27	1	0	3.852550	1.354930	-0.028228
28	1	0	3.539129	-0.025181	1.015644
29	1	0	-0.134480	0.843761	0.137401
30	1	0	-1.777918	-1.509263	-1.367155
31	8	0	-2.019835	-0.232176	-3.020698
32	1	0	-2.892312	-0.125778	-3.414953
33	1	0	-1.802183	0.629945	-2.611491
34	8	0	0.875868	3.057205	1.794535
35	1	0	1.337206	3.892098	1.655788
36	1	0	1.377978	2.353521	1.280498
37	8	0	-1.254975	2.172091	-1.877030
38	1	0	-1.399804	2.950344	-2.426929
39	1	0	-1.430151	2.451269	-0.951686
40	8	0	-1.582278	2.941685	0.721679
41	1	0	-2.201782	2.532440	1.335979
42	1	0	-0.714398	3.025035	1.187932

(S,R)_4H2O_neutral_R

Energy: -806.519458 hartree

Center Number	Atomic Number	Atomic Type	Coordinates (Angstroms) X	Y	Z
1	6	0	-2.654074	-0.311234	0.181769
2	6	0	-1.370531	-1.264182	-1.582965
3	6	0	-1.050015	-2.526032	-0.831146
4	6	0	-1.953801	-2.705325	0.386795
5	6	0	-2.067055	-1.367622	1.115484
6	6	0	1.596856	0.387968	-1.229963

7	6	0	2.984302	-0.078258	-1.599830
8	6	0	3.792446	-0.364439	-0.328903
9	6	0	2.981449	-1.253295	0.617937
10	7	0	-2.079334	-0.290595	-1.169306
11	7	0	1.693428	-0.654357	0.940812
12	6	0	1.088811	0.143461	-0.003541
13	1	0	-2.512384	0.691583	0.599899
14	1	0	-3.735458	-0.458206	0.064905
15	1	0	-1.114534	-3.372010	-1.523109
16	1	0	0.004691	-2.451046	-0.525045
17	1	0	-2.949311	-3.032005	0.061277
18	1	0	-1.555713	-3.482770	1.044608
19	1	0	-2.697628	-1.449544	2.006253
20	1	0	-1.070552	-1.054847	1.452764
21	1	0	-0.936248	-1.159775	-2.580907
22	1	0	0.990970	0.921753	-1.957433
23	1	0	2.948768	-0.981472	-2.225247
24	1	0	3.490722	0.687398	-2.198565
25	1	0	4.741442	-0.852209	-0.570348
26	1	0	4.021385	0.580364	0.178707
27	1	0	3.523153	-1.438463	1.548525
28	1	0	2.817391	-2.227048	0.132969
29	1	0	0.119875	0.541344	0.296947
30	1	0	-1.873244	1.197742	-1.943495
31	8	0	-1.573727	2.080821	-2.320750
32	1	0	-2.367581	2.562042	-2.580065
33	1	0	-0.564540	2.845020	-1.145621
34	8	0	0.286744	0.456467	3.295986
35	1	0	0.776837	0.903437	3.994758
36	1	0	1.464916	-0.444719	1.906921
37	8	0	0.018013	3.182334	-0.423525
38	1	0	0.841358	2.678697	-0.506589
39	1	0	-0.725500	2.714141	1.067443
40	8	0	-1.144672	2.340985	1.878450
41	1	0	-1.502277	3.086409	2.373884
42	1	0	-0.216388	1.157728	2.830905

(S,R)_4H2O_neutral_TS

Energy: -806.484824 hartree

Center Number	Atomic Number	Atomic Type	Coordinates (Angstroms) X	Y	Z

1	6	0	-1.946065	-0.682630	1.167954
2	6	0	0.040873	-1.909532	0.365623
3	6	0	0.836899	-1.620466	1.619170
4	6	0	-0.066405	-1.130709	2.754389
5	6	0	-1.018097	-0.066655	2.212120
6	6	0	0.967645	-0.716293	-1.239780
7	6	0	2.388087	-1.223946	-1.385736
8	6	0	3.378010	-0.051921	-1.330490
9	6	0	3.134782	0.803040	-0.088656
10	7	0	-1.270637	-1.714696	0.375251
11	7	0	1.726293	1.186937	-0.005520
12	6	0	0.769320	0.517503	-0.650755
13	1	0	-2.316317	0.087611	0.480732
14	1	0	-2.807482	-1.156253	1.650468
15	1	0	1.362374	-2.539364	1.897374
16	1	0	1.603831	-0.867896	1.415698
17	1	0	-0.642406	-1.970042	3.162108
18	1	0	0.548994	-0.731174	3.564239
19	1	0	-1.627954	0.367348	3.009024
20	1	0	-0.441106	0.754122	1.769640
21	1	0	0.368002	-2.736870	-0.256516
22	1	0	0.202906	-1.052772	-1.931986
23	1	0	2.638213	-1.941046	-0.588765
24	1	0	2.499189	-1.764480	-2.329972
25	1	0	4.410385	-0.410701	-1.317957
26	1	0	3.250633	0.570369	-2.223271
27	1	0	3.733890	1.716491	-0.118901
28	1	0	3.428341	0.249146	0.811845
29	1	0	-0.219549	0.976919	-0.625493
30	1	0	-1.801369	-2.021285	-0.444178
31	8	0	-2.735551	-1.648171	-2.048973
32	1	0	-3.679240	-1.823278	-2.127572
33	1	0	-2.626000	-0.656798	-2.050524
34	8	0	0.109440	3.238329	1.015701
35	1	0	0.243109	4.170493	0.814687
36	1	0	1.435181	2.054192	0.455695
37	8	0	-2.304243	0.946034	-1.990559
38	1	0	-2.517564	1.444044	-2.786483
39	1	0	-2.201393	1.646525	-1.173334
40	8	0	-1.996153	2.507536	-0.127542
41	1	0	-2.750789	2.513922	0.471645
42	1	0	-0.781691	2.954922	0.551627

2.2 Acidic conditions

(R,S)_2H2O_Green_F
Energy: -654.188437 hartree

Center Number	Atomic Number	Atomic Type	Coordinates (Angstroms) X	Y	Z
1	6	0	-2.261411	-1.520472	0.755289
2	6	0	-1.169455	0.150255	-0.618143
3	6	0	-2.458193	0.968924	-0.703530
4	6	0	-3.681284	0.059046	-0.570599
5	6	0	-3.585953	-0.766022	0.714194
6	6	0	0.079847	1.054586	-0.747807
7	6	0	0.176093	2.170671	0.298430
8	6	0	1.610174	2.686299	0.412185
9	6	0	2.546167	1.560294	0.818485
10	7	0	-1.135280	-0.585756	0.645902
11	7	0	2.335313	0.390164	-0.053286
12	6	0	1.284545	0.178887	-0.754065
13	1	0	-2.246330	-2.259381	-0.067465
14	1	0	-2.156929	-2.070977	1.694679
15	1	0	-2.466918	1.703058	0.111403
16	1	0	-2.479474	1.519164	-1.650954
17	1	0	-4.598868	0.655512	-0.582955
18	1	0	-3.725442	-0.618548	-1.434067
19	1	0	-3.645069	-0.100784	1.584781
20	1	0	-4.416857	-1.475603	0.781720
21	1	0	-1.155923	-0.547918	-1.478877
22	1	0	0.042775	1.485362	-1.760927
23	1	0	-0.495607	2.985854	0.021196
24	1	0	-0.158916	1.778239	1.264641
25	1	0	1.940664	3.103211	-0.546001
26	1	0	1.678530	3.481375	1.157683
27	1	0	2.369285	1.232028	1.847130
28	1	0	3.596871	1.836100	0.721031
29	1	0	1.283758	-0.724689	-1.362424
30	1	0	-0.280541	-1.141541	0.687883
31	8	0	3.791761	-2.034126	-0.322428
32	1	0	3.078555	-0.321307	-0.105244
33	1	0	4.181954	-2.248251	-1.180185
34	8	0	1.059257	-2.663430	0.077693
35	1	0	0.722897	-3.564586	0.140272
36	1	0	2.025534	-2.733941	0.002302
37	1	0	4.454495	-2.277571	0.337322

(R,S)_2H2O_Green_R

Energy: -654.172244 hartree

Center Number	Atomic Number	Atomic Type	Coordinates (Angstroms) X	Y	Z
1	6	0	-1.521549	-1.207197	1.414556
2	6	0	-1.217482	-1.065552	-1.008895
3	6	0	-2.530845	-0.406201	-1.241830
4	6	0	-3.438923	-0.466929	-0.013331
5	6	0	-2.624499	-0.172306	1.245770
6	6	0	0.537275	1.542948	-1.499973
7	6	0	-0.100110	2.642571	-0.687917
8	6	0	0.712787	2.871885	0.591437
9	6	0	0.937317	1.544747	1.317549
10	7	0	-0.804015	-1.425891	0.149223
11	7	0	1.542125	0.539852	0.446250
12	6	0	1.360708	0.648608	-0.917607
13	1	0	-1.925651	-2.175560	1.725493
14	1	0	-0.773165	-0.903031	2.149434
15	1	0	-2.311361	0.630114	-1.533216
16	1	0	-2.985731	-0.873403	-2.120670
17	1	0	-4.254701	0.250050	-0.123430
18	1	0	-3.886899	-1.463750	0.062716
19	1	0	-2.184175	0.829342	1.171698
20	1	0	-3.254637	-0.186865	2.137653
21	1	0	-0.559043	-1.294393	-1.842322
22	1	0	0.400719	1.509532	-2.575941
23	1	0	-0.142342	3.565434	-1.276291
24	1	0	-1.142999	2.405945	-0.424292
25	1	0	1.683777	3.307749	0.328653
26	1	0	0.202839	3.569372	1.262045
27	1	0	-0.032363	1.177877	1.681855
28	1	0	1.578331	1.677299	2.192317
29	1	0	1.898208	-0.095118	-1.502454
30	1	0	0.125554	-1.883710	0.174702
31	8	0	3.922276	-1.283121	0.296306
32	1	0	2.424119	0.135308	0.734922
33	1	0	4.574672	-1.090137	-0.389017
34	8	0	1.595326	-2.599391	-0.489892
35	1	0	1.695758	-3.558314	-0.488116

36	1	0	2.452309	-2.226177	-0.205598
37	1	0	4.421129	-1.640644	1.041680

(R,S)_2H2O_Green_TS

Energy: -654.157719 hartree

Center Number	Atomic Number	Atomic Type	Coordinates (Angstroms) X	Y	Z
1	6	0	-1.647607	-0.695143	1.585297
2	6	0	-0.934002	-0.777127	-0.806998
3	6	0	-2.334755	-0.441418	-1.282159
4	6	0	-3.361107	-0.918834	-0.249200
5	6	0	-3.035165	-0.269377	1.093408
6	6	0	0.091792	1.068586	-1.393610
7	6	0	-0.507094	2.147342	-0.514050
8	6	0	0.570439	2.782470	0.371929
9	6	0	1.340476	1.698934	1.119553
10	7	0	-0.737093	-1.012103	0.485014
11	7	0	1.857218	0.713748	0.168229
12	6	0	1.324816	0.552223	-1.042041
13	1	0	-1.724127	-1.604002	2.190116
14	1	0	-1.204615	0.079721	2.221766
15	1	0	-2.461558	0.635148	-1.425302
16	1	0	-2.479964	-0.913358	-2.256988
17	1	0	-4.365747	-0.638515	-0.575111
18	1	0	-3.332921	-2.011138	-0.162270
19	1	0	-3.083061	0.819327	0.980034
20	1	0	-3.768805	-0.540569	1.856530
21	1	0	-0.305975	-1.377371	-1.455187
22	1	0	-0.179277	1.020881	-2.443545
23	1	0	-0.987639	2.911319	-1.131715
24	1	0	-1.292423	1.736963	0.136496
25	1	0	1.268694	3.354976	-0.248419
26	1	0	0.124269	3.468121	1.096320
27	1	0	0.688241	1.204002	1.849007
28	1	0	2.189093	2.118123	1.664467
29	1	0	1.869211	-0.115218	-1.705497
30	1	0	0.129334	-1.500526	0.708223
31	8	0	4.103636	-1.204254	0.179088
32	1	0	2.728247	0.232783	0.373482
33	1	0	4.720356	-1.173189	-0.563444

34	8	0	1.618058	-2.478548	-0.146846
35	1	0	1.626732	-3.442268	-0.162934
36	1	0	2.542767	-2.197505	-0.027933
37	1	0	4.637987	-1.402729	0.958481

(R,S)_2H2O_Red_F

Energy: -654.186186 hartree

Center Number	Atomic Number	Atomic Type	Coordinates (Angstroms) X	Y	Z
1	6	0	2.170809	-1.318064	-1.036291
2	6	0	1.092009	0.053814	0.645025
3	6	0	2.428829	0.689905	1.031745
4	6	0	3.587083	-0.265590	0.735797
5	6	0	3.538046	-0.721168	-0.723273
6	6	0	-0.093500	1.016782	0.919155
7	6	0	-0.167122	2.269749	0.029498
8	6	0	-1.073829	2.063797	-1.184724
9	6	0	-2.467881	1.648468	-0.747475
10	7	0	1.110549	-0.340725	-0.761701
11	7	0	-2.381394	0.515347	0.190307
12	6	0	-1.354576	0.228437	0.900454
13	1	0	2.033938	-2.233037	-0.430315
14	1	0	2.103591	-1.605928	-2.089361
15	1	0	2.572273	1.614754	0.460368
16	1	0	2.407197	0.954790	2.094540
17	1	0	4.542047	0.219204	0.961418
18	1	0	3.508975	-1.143816	1.391016
19	1	0	3.714175	0.136259	-1.384981
20	1	0	4.318853	-1.461561	-0.924638
21	1	0	0.948520	-0.829741	1.299096
22	1	0	0.017418	1.313129	1.972789
23	1	0	-0.548032	3.106526	0.624212
24	1	0	0.837003	2.539639	-0.302606
25	1	0	-1.153069	2.987999	-1.761673
26	1	0	-0.651534	1.294795	-1.838313
27	1	0	-3.084493	1.317134	-1.583376
28	1	0	-2.992931	2.457163	-0.230162
29	1	0	-1.430608	-0.661842	1.521329
30	1	0	0.222757	-0.794353	-0.977593
31	8	0	-3.827818	-1.911537	0.464917

32	1	0	-4.092849	-2.218682	1.341856
33	1	0	-3.180792	-0.126629	0.262676
34	8	0	-1.138505	-2.366005	-0.348247
35	1	0	-0.780643	-3.250651	-0.484734
36	1	0	-2.083163	-2.481053	-0.150878
37	1	0	-4.561341	-2.132899	-0.123731

(R,S)_2H2O_Red_R

Energy: -654.172852 hartree

Center	Atomic	Atomic	Coordinates (Angstroms)		
Number	Number	Type	X	Y	Z
1	6	0	1.741143	-0.875978	-1.515436
2	6	0	1.156677	-1.100314	0.853116
3	6	0	2.469541	-0.592248	1.332944
4	6	0	3.523072	-0.583094	0.225423
5	6	0	2.914648	-0.019558	-1.057943
6	6	0	-0.622181	1.502218	1.521762
7	6	0	0.041076	2.593041	0.715793
8	6	0	-0.151323	2.360392	-0.789290
9	6	0	-1.561969	1.863218	-1.093074
10	7	0	0.864292	-1.229541	-0.387734
11	7	0	-1.784632	0.603259	-0.390913
12	6	0	-1.467315	0.617087	0.961326
13	1	0	2.080458	-1.817051	-1.958607
14	1	0	1.116480	-0.364314	-2.251179
15	1	0	2.288411	0.417786	1.727161
16	1	0	2.768621	-1.199851	2.191964
17	1	0	4.381937	0.012993	0.539233
18	1	0	3.879046	-1.604458	0.051023
19	1	0	2.579953	1.008842	-0.880114
20	1	0	3.651129	0.013140	-1.863746
21	1	0	0.387837	-1.416383	1.552679
22	1	0	-0.409896	1.421406	2.583567
23	1	0	-0.364331	3.573448	0.999137
24	1	0	1.113700	2.637786	0.947018
25	1	0	0.054079	3.275165	-1.352328
26	1	0	0.553335	1.598701	-1.139737
27	1	0	-1.692974	1.696322	-2.165015
28	1	0	-2.292822	2.623096	-0.772381
29	1	0	-1.915954	-0.184420	1.544592

30	1	0	-0.072758	-1.616143	-0.579688
31	8	0	-4.025264	-1.405768	-0.151222
32	1	0	-4.581644	-1.347682	0.635757
33	1	0	-2.665230	0.143125	-0.594100
34	8	0	-1.493275	-2.544534	0.153402
35	1	0	-1.518147	-3.507521	0.115969
36	1	0	-2.411687	-2.237523	0.031419
37	1	0	-4.599076	-1.728654	-0.857255

(R,S)_2H2O_Red_TS

Energy: -654.154887 hartree

Center Number	Atomic Number	Atomic Type	Coordinates (Angstroms) X	Y	Z
1	6	0	1.635011	-0.880464	-1.581379
2	6	0	0.905184	-0.804736	0.811796
3	6	0	2.322748	-0.524751	1.294745
4	6	0	3.359273	-0.830164	0.210366
5	6	0	2.909347	-0.197064	-1.103850
6	6	0	-0.060967	1.009794	1.420323
7	6	0	0.515050	2.189993	0.671609
8	6	0	0.032095	2.216224	-0.784107
9	6	0	-1.478745	2.036186	-0.878240
10	7	0	0.705036	-1.105449	-0.475883
11	7	0	-1.907401	0.878379	-0.091517
12	6	0	-1.315660	0.547620	1.049325
13	1	0	1.881359	-1.860969	-2.005722
14	1	0	1.138400	-0.302830	-2.367722
15	1	0	2.434916	0.514873	1.609199
16	1	0	2.486243	-1.136918	2.186429
17	1	0	4.333333	-0.443504	0.519998
18	1	0	3.462403	-1.913853	0.078199
19	1	0	2.735864	0.876250	-0.952524
20	1	0	3.671084	-0.295126	-1.881280
21	1	0	0.272595	-1.379967	1.478885
22	1	0	0.214603	0.873594	2.462294
23	1	0	0.220652	3.119937	1.174547
24	1	0	1.611096	2.173876	0.695086
25	1	0	0.309232	3.160185	-1.260479
26	1	0	0.512311	1.414997	-1.350281
27	1	0	-1.789431	1.876444	-1.912109

28	1	0	-1.997218	2.926602	-0.502454
29	1	0	-1.818708	-0.225202	1.626049
30	1	0	-0.173166	-1.579885	-0.678367
31	8	0	-4.157039	-1.017954	-0.249156
32	1	0	-4.798059	-1.003710	0.473083
33	1	0	-2.789037	0.424822	-0.317713
34	8	0	-1.755520	-2.426652	0.142920
35	1	0	-1.813329	-3.388677	0.160910
36	1	0	-2.657998	-2.100370	-0.023066
37	1	0	-4.670134	-1.160061	-1.054800

(R,S)_3H2O_Green_F

Energy: -730.580481 hartree

Center Number	Atomic Number	Atomic Type	Coordinates (Angstroms) X	Y	Z
1	6	0	-2.418688	-1.836225	0.387202
2	6	0	-1.645170	0.224655	-0.627285
3	6	0	-3.007857	0.882339	-0.403636
4	6	0	-4.117601	-0.171147	-0.391187
5	6	0	-3.804475	-1.253743	0.643519
6	6	0	-0.510480	1.275843	-0.636550
7	6	0	-0.394528	2.110838	0.643522
8	6	0	0.976904	2.778815	0.733711
9	6	0	2.074946	1.728516	0.734980
10	7	0	-1.404472	-0.776860	0.410311
11	7	0	1.878420	0.788891	-0.384188
12	6	0	0.767822	0.596574	-0.991250
13	1	0	-2.428175	-2.359262	-0.587323
14	1	0	-2.159194	-2.572772	1.153222
15	1	0	-2.997031	1.402643	0.561950
16	1	0	-3.185670	1.629577	-1.185291
17	1	0	-5.083221	0.299801	-0.182064
18	1	0	-4.192283	-0.631613	-1.385741
19	1	0	-3.828772	-0.819465	1.650975
20	1	0	-4.553069	-2.051825	0.607934
21	1	0	-1.660710	-0.244548	-1.631595
22	1	0	-0.713143	1.933960	-1.497026
23	1	0	-1.181854	2.867605	0.654005
24	1	0	-0.551297	1.457177	1.508253
25	1	0	1.125952	3.456067	-0.114944

26	1	0	1.060525	3.372047	1.646748
27	1	0	2.072815	1.137658	1.656259
28	1	0	3.067475	2.163330	0.607613
29	1	0	0.783067	-0.123041	-1.807133
30	1	0	-0.496546	-1.218394	0.256820
31	8	0	3.386248	-2.082106	1.195334
32	1	0	3.968293	-1.521852	0.654264
33	1	0	3.344314	-1.680524	2.070638
34	8	0	4.374761	-0.520422	-0.919458
35	1	0	5.134944	0.076095	-0.924696
36	1	0	2.710260	0.263178	-0.692183
37	8	0	1.181026	-2.207795	-0.463916
38	1	0	1.003494	-3.111937	-0.746624
39	1	0	1.908084	-2.262095	0.189024
40	1	0	4.472597	-1.082230	-1.699790

(R,S)_3H2O_Green_R

Energy: -730.565311 hartree

Center Number	Atomic Number	Atomic Type	Coordinates (Angstroms) X	Y	Z
1	6	0	-1.391352	-1.636319	0.991727
2	6	0	-1.879736	-0.640608	-1.183963
3	6	0	-3.180306	-0.064646	-0.743923
4	6	0	-3.638446	-0.613672	0.607270
5	6	0	-2.447354	-0.701026	1.560880
6	6	0	-0.359052	2.140837	-1.254607
7	6	0	-0.631025	2.797521	0.075898
8	6	0	0.593559	2.646174	0.984868
9	6	0	1.037448	1.182855	1.017299
10	7	0	-1.120907	-1.337998	-0.423779
11	7	0	1.291134	0.650858	-0.319722
12	6	0	0.612248	1.216056	-1.384061
13	1	0	-1.713145	-2.681340	1.043121
14	1	0	-0.435535	-1.554989	1.514907
15	1	0	-3.043329	1.024925	-0.714318
16	1	0	-3.909702	-0.250910	-1.538438
17	1	0	-4.419698	0.027970	1.019275
18	1	0	-4.071768	-1.610488	0.470143
19	1	0	-2.017957	0.297538	1.708155
20	1	0	-2.751604	-1.073325	2.541526

21	1	0	-1.544792	-0.499658	-2.207445
22	1	0	-0.894325	2.467562	-2.140480
23	1	0	-0.860587	3.858118	-0.073208
24	1	0	-1.513915	2.365790	0.572736
25	1	0	1.412776	3.262356	0.595868
26	1	0	0.372968	2.984107	2.001446
27	1	0	0.247743	0.589530	1.499150
28	1	0	1.943773	1.053958	1.615197
29	1	0	0.885044	0.812343	-2.356691
30	1	0	-0.226101	-1.722697	-0.811604
31	8	0	2.984273	-2.155178	0.756699
32	1	0	3.522097	-1.346420	0.656594
33	1	0	2.562729	-2.097550	1.622130
34	8	0	4.293080	0.211363	0.121647
35	1	0	4.663681	0.806128	0.785612
36	1	0	2.253801	0.390739	-0.500291
37	8	0	1.223364	-2.398856	-1.308108
38	1	0	1.204477	-3.331796	-1.552173
39	1	0	1.872784	-2.313775	-0.572438
40	1	0	4.967354	0.130193	-0.564493

(R,S)_3H2O_Green_TS
Energy: -730.550155 hartree

Center Number	Atomic Number	Atomic Type	Coordinates (Angstroms) X	Y	Z
1	6	0	-1.649855	-0.950026	1.620148
2	6	0	-1.380809	-0.740619	-0.852902
3	6	0	-2.868045	-0.513543	-1.042971
4	6	0	-3.644997	-1.177219	0.099033
5	6	0	-3.132672	-0.618132	1.424042
6	6	0	-0.641663	1.251949	-1.414794
7	6	0	-1.182090	2.189140	-0.353715
8	6	0	-0.033973	2.872104	0.397472
9	6	0	0.950642	1.824709	0.906568
10	7	0	-0.931013	-1.067127	0.351645
11	7	0	1.388441	0.972741	-0.200235
12	6	0	0.675772	0.846231	-1.319032
13	1	0	-1.542924	-1.916297	2.123044
14	1	0	-1.161847	-0.200556	2.254026
15	1	0	-3.111446	0.552310	-1.055995

16	1	0	-3.146143	-0.917004	-2.019793
17	1	0	-4.711843	-0.970733	-0.015986
18	1	0	-3.512053	-2.264805	0.066989
19	1	0	-3.286181	0.466668	1.433344
20	1	0	-3.691091	-1.023988	2.271132
21	1	0	-0.828931	-1.204282	-1.662321
22	1	0	-1.083647	1.252993	-2.406123
23	1	0	-1.834423	2.938146	-0.811599
24	1	0	-1.799005	1.647141	0.377360
25	1	0	0.490112	3.560485	-0.274604
26	1	0	-0.411652	3.450310	1.244240
27	1	0	0.481943	1.210385	1.684787
28	1	0	1.838995	2.289414	1.341100
29	1	0	1.158529	0.294789	-2.120536
30	1	0	0.000554	-1.482961	0.362680
31	8	0	3.960313	-2.151227	0.131994
32	1	0	4.190577	-1.228566	0.348945
33	1	0	4.161329	-2.676740	0.914595
34	8	0	4.267000	0.595238	0.516804
35	1	0	4.431801	0.923850	1.409895
36	1	0	2.344593	0.632695	-0.169638
37	8	0	1.378478	-2.228072	-0.824565
38	1	0	1.214880	-3.102006	-1.197272
39	1	0	2.297787	-2.248477	-0.484840
40	1	0	4.907902	1.042865	-0.050261

(R,S)_3H2O_Red_F

Energy: -730.568688 hartree

Center Number	Atomic Number	Atomic Type	Coordinates (Angstroms) X	Y	Z
1	6	0	2.258170	1.160247	0.855449
2	6	0	1.438919	-0.314415	-0.938086
3	6	0	2.896775	-0.799017	-0.891051
4	6	0	3.841454	0.400979	-0.968013
5	6	0	3.678713	1.291193	0.280909
6	6	0	0.459708	-1.440708	-0.456699
7	6	0	0.532459	-1.858905	1.020571
8	6	0	-0.364506	-0.995383	1.910669
9	6	0	-1.806014	-1.063489	1.437325
10	7	0	1.313532	0.963008	-0.241740

11	7	0	-1.877303	-0.863301	-0.019351
12	6	0	-0.912628	-1.029850	-0.844896
13	1	0	1.970648	2.070321	1.389314
14	1	0	2.231572	0.342117	1.593761
15	1	0	3.094849	-1.352845	0.034507
16	1	0	3.063445	-1.494795	-1.718584
17	1	0	4.877217	0.063894	-1.067482
18	1	0	3.604413	0.974914	-1.870902
19	1	0	4.403811	1.003562	1.049967
20	1	0	3.880042	2.334654	0.018936
21	1	0	1.192726	-0.141154	-1.996236
22	1	0	0.691924	-2.301409	-1.101093
23	1	0	0.212044	-2.903376	1.102377
24	1	0	1.565697	-1.824386	1.371560
25	1	0	-0.323266	-1.347048	2.944175
26	1	0	-0.042616	0.049775	1.899726
27	1	0	-2.428396	-0.285179	1.880465
28	1	0	-2.262150	-2.036528	1.645199
29	1	0	-1.143269	-0.817497	-1.887835
30	1	0	0.358570	1.191503	0.025063
31	8	0	-3.977854	1.095538	0.350018
32	1	0	-4.755314	1.369420	0.850285
33	1	0	-4.223463	1.087188	-0.589679
34	8	0	-3.482822	0.220128	-2.200425
35	1	0	-4.063344	-0.334612	-2.736647
36	1	0	-2.768491	-0.536851	-0.403625
37	8	0	-1.371394	1.990540	0.857580
38	1	0	-1.264707	2.933835	1.024435
39	1	0	-2.321657	1.850898	0.688818
40	1	0	-3.085638	0.852090	-2.813321

(R,S)_3H2O_Red_R

Energy: -730.566735 hartree

Center Number	Atomic Number	Atomic Type	Coordinates (Angstroms) X	Y	Z
1	6	0	-1.823441	-1.542598	0.987975
2	6	0	-1.832076	-0.482681	-1.212849
3	6	0	-3.154634	0.169837	-1.006500
4	6	0	-3.901818	-0.394218	0.201596
5	6	0	-2.932174	-0.572235	1.368804

6	6	0	-0.202958	2.253444	-0.979652
7	6	0	-0.457221	2.597774	0.467917
8	6	0	0.259530	1.611791	1.401179
9	6	0	1.652363	1.274761	0.875766
10	7	0	-1.270607	-1.235098	-0.341906
11	7	0	1.531841	0.665224	-0.443984
12	6	0	0.736734	1.361536	-1.344773
13	1	0	-2.189596	-2.573325	0.947789
14	1	0	-0.986117	-1.513716	1.689527
15	1	0	-2.957243	1.244800	-0.893308
16	1	0	-3.723594	0.067449	-1.935575
17	1	0	-4.718486	0.276699	0.474816
18	1	0	-4.345802	-1.360889	-0.060039
19	1	0	-2.500047	0.398737	1.639295
20	1	0	-3.443217	-0.958936	2.253111
21	1	0	-1.303444	-0.352687	-2.152597
22	1	0	-0.796103	2.732177	-1.753019
23	1	0	-0.125725	3.623124	0.678571
24	1	0	-1.535519	2.587946	0.678588
25	1	0	0.327738	2.016771	2.414700
26	1	0	-0.308218	0.676801	1.462089
27	1	0	2.157628	0.570124	1.542152
28	1	0	2.257423	2.195159	0.827694
29	1	0	0.893247	1.104554	-2.389767
30	1	0	-0.350602	-1.682130	-0.583129
31	8	0	3.156321	-2.078229	0.702501
32	1	0	2.967306	-2.068419	1.647647
33	1	0	3.702244	-1.290715	0.513945
34	8	0	4.514544	0.187903	-0.155836
35	1	0	4.829030	0.861157	0.460734
36	1	0	2.419544	0.352696	-0.820319
37	8	0	1.051696	-2.470769	-0.986118
38	1	0	1.004964	-3.431464	-1.058466
39	1	0	1.795999	-2.273328	-0.373194
40	1	0	5.253617	0.010824	-0.751157

(R,S)_3H2O_Red_TS

Energy: -730.547954 hartree

Center Number	Atomic Number	Atomic Type	Coordinates (Angstroms) X	Y	Z

1	6	0	2.261183	1.130634	1.245994
2	6	0	1.564398	0.245167	-0.986697
3	6	0	2.935682	-0.369936	-1.205157
4	6	0	3.993789	0.422923	-0.432841
5	6	0	3.600352	0.417838	1.041820
6	6	0	0.394654	-1.581463	-0.841517
7	6	0	0.559550	-2.000001	0.601854
8	6	0	-0.459091	-1.285483	1.502867
9	6	0	-1.866863	-1.378168	0.926952
10	7	0	1.381055	1.012862	0.081891
11	7	0	-1.870671	-0.898527	-0.451921
12	6	0	-0.844454	-1.128000	-1.266393
13	1	0	2.426907	2.202831	1.393309
14	1	0	1.750702	0.755467	2.139386
15	1	0	2.965673	-1.403874	-0.849846
16	1	0	3.130049	-0.393456	-2.280263
17	1	0	4.972802	-0.042440	-0.571347
18	1	0	4.056959	1.450157	-0.810274
19	1	0	3.535436	-0.620776	1.385882
20	1	0	4.352547	0.912325	1.661437
21	1	0	1.010681	0.576876	-1.857122
22	1	0	1.007904	-2.048945	-1.604758
23	1	0	0.442361	-3.086065	0.702142
24	1	0	1.575652	-1.772777	0.951809
25	1	0	-0.447375	-1.715310	2.507878
26	1	0	-0.201872	-0.225600	1.592280
27	1	0	-2.567519	-0.766735	1.499659
28	1	0	-2.225587	-2.415180	0.947673
29	1	0	-1.012489	-0.876812	-2.310990
30	1	0	0.554443	1.613182	0.041425
31	8	0	-3.248593	1.980717	0.727283
32	1	0	-3.162934	1.934643	1.686075
33	1	0	-3.877257	1.284980	0.461276
34	8	0	-4.712019	-0.097933	-0.419750
35	1	0	-5.227093	-0.740878	0.084214
36	1	0	-2.746561	-0.542093	-0.819337
37	8	0	-0.995761	2.223069	-0.848584
38	1	0	-0.999077	3.149939	-1.113823
39	1	0	-1.791504	2.102360	-0.289637
40	1	0	-5.279761	0.179961	-1.149730

(R,S)_4H2O_Red_F

Energy: -806.961702 hartree

Center Number	Atomic Number	Atomic Type	Coordinates (Angstroms) X	Y	Z
1	6	0	2.197369	1.217323	0.155445
2	6	0	1.595144	-1.218669	0.044086
3	6	0	1.629486	-1.326950	1.594445
4	6	0	2.443850	-0.196725	2.230736
5	6	0	1.961624	1.142863	1.676537
6	6	0	0.139393	-1.258370	-0.530868
7	6	0	-0.476043	-2.656443	-0.413620
8	6	0	-1.986737	-2.608747	-0.630631
9	6	0	-2.630802	-1.723392	0.423874
10	7	0	2.427439	-0.116374	-0.413798
11	7	0	-1.918368	-0.436021	0.511464
12	6	0	-0.729851	-0.217470	0.078094
13	1	0	3.101013	1.802644	-0.045106
14	1	0	1.369076	1.756271	-0.332050
15	1	0	0.609088	-1.280936	2.001118
16	1	0	2.028861	-2.300478	1.891741
17	1	0	2.324138	-0.226742	3.318011
18	1	0	3.508848	-0.329698	2.013900
19	1	0	0.893243	1.250547	1.909820
20	1	0	2.464326	1.980314	2.168328
21	1	0	2.049267	-2.123109	-0.377327
22	1	0	0.216514	-0.968526	-1.590640
23	1	0	0.001805	-3.316858	-1.141607
24	1	0	-0.264660	-3.066874	0.582303
25	1	0	-2.211317	-2.211462	-1.627111
26	1	0	-2.426657	-3.606190	-0.565397
27	1	0	-2.593660	-2.182729	1.416474
28	1	0	-3.671216	-1.490769	0.192868
29	1	0	-0.372998	0.804804	0.186463
30	1	0	2.396549	-0.057297	-1.429475
31	8	0	-0.887818	1.471479	-2.247732
32	1	0	-0.946927	2.172820	-1.569394
33	1	0	-1.614456	1.616806	-2.864523
34	8	0	-3.032721	2.008361	1.539446
35	1	0	-3.021797	2.115407	2.499959
36	1	0	-2.423160	0.358238	0.930708
37	8	0	1.495233	0.466363	-3.247628
38	1	0	1.906955	1.004424	-3.932485
39	1	0	0.683988	0.938197	-2.975989
40	8	0	-0.870881	2.969171	0.056512

41	1	0	-1.660004	2.913809	0.624488
42	1	0	-0.472462	3.835264	0.199958
43	1	0	-3.928943	2.235371	1.257925

(R,S)_4H2O_Red_R

Energy: -806.959229 hartree

Center Number	Atomic Number	Atomic Type	Coordinates (Angstroms) X	Y	Z
1	6	0	2.514345	-0.942215	-0.101613
2	6	0	0.770895	-1.016593	-1.816453
3	6	0	0.186482	-2.286649	-1.315885
4	6	0	1.082941	-2.986885	-0.294449
5	6	0	1.671565	-1.951479	0.663996
6	6	0	-1.440813	1.075181	-0.925687
7	6	0	-2.823383	0.842281	-1.483200
8	6	0	-3.758184	0.373592	-0.362341
9	6	0	-3.128434	-0.798509	0.392851
10	7	0	1.801495	-0.459294	-1.297652
11	7	0	-1.797388	-0.463993	0.892119
12	6	0	-1.057338	0.490087	0.227151
13	1	0	3.458212	-1.378220	-0.442918
14	1	0	2.729798	-0.059439	0.504789
15	1	0	-0.778204	-2.007978	-0.865333
16	1	0	-0.046484	-2.916688	-2.179753
17	1	0	0.501553	-3.734241	0.249436
18	1	0	1.892325	-3.513711	-0.811984
19	1	0	0.858779	-1.430267	1.185166
20	1	0	2.299673	-2.424580	1.422365
21	1	0	0.320516	-0.507059	-2.665024
22	1	0	-0.753641	1.741910	-1.438306
23	1	0	-3.207205	1.765573	-1.930341
24	1	0	-2.807667	0.093881	-2.288690
25	1	0	-3.930001	1.200415	0.336758
26	1	0	-4.728495	0.067762	-0.763637
27	1	0	-3.058820	-1.659789	-0.287683
28	1	0	-3.749693	-1.103365	1.238023
29	1	0	-0.089686	0.702291	0.679967
30	1	0	2.091082	0.464687	-1.680748
31	8	0	0.997054	3.147784	0.219042
32	1	0	1.258844	2.566245	0.964112

33	1	0	0.030969	3.144793	0.204011
34	8	0	-0.184878	-0.137903	3.415696
35	1	0	0.135694	-0.952488	3.824759
36	1	0	-1.611956	-0.551504	1.882980
37	8	0	2.075168	2.144696	-2.063859
38	1	0	2.886592	2.598251	-2.318807
39	1	0	1.723609	2.601548	-1.265029
40	8	0	1.796681	1.357646	2.154940
41	1	0	1.141986	0.864655	2.688464
42	1	0	2.475784	1.663706	2.767622
43	1	0	-0.652970	0.335969	4.115764

(R,S)_4H2O_Red_TS

Energy: -806.944130 hartree

Center Number	Atomic Number	Atomic Type	Coordinates (Angstroms) X	Y	Z
1	6	0	1.972020	-0.427013	1.402183
2	6	0	0.032666	-1.790992	0.696376
3	6	0	-0.808814	-1.371056	1.886337
4	6	0	0.052266	-0.727446	2.977000
5	6	0	0.995022	0.287490	2.335132
6	6	0	-0.832819	-0.885942	-1.029294
7	6	0	-2.220613	-1.483401	-1.171341
8	6	0	-3.264286	-0.374396	-1.356352
9	6	0	-3.140339	0.670100	-0.250791
10	7	0	1.347523	-1.574452	0.738767
11	7	0	-1.756415	1.143879	-0.161316
12	6	0	-0.734079	0.440228	-0.642468
13	1	0	2.827866	-0.805417	1.970320
14	1	0	2.350710	0.262064	0.637508
15	1	0	-1.586909	-0.666624	1.578394
16	1	0	-1.321650	-2.261470	2.262320
17	1	0	-0.592985	-0.249431	3.717928
18	1	0	0.636578	-1.496402	3.495679
19	1	0	0.404078	1.026992	1.780453
20	1	0	1.565690	0.837714	3.087312
21	1	0	-0.240444	-2.720581	0.204880
22	1	0	-0.027498	-1.261843	-1.652265
23	1	0	-2.244592	-2.169429	-2.022124
24	1	0	-2.486217	-2.078497	-0.285221

25	1	0	-3.112097	0.111268	-2.326406
26	1	0	-4.276994	-0.784354	-1.342472
27	1	0	-3.449398	0.244541	0.711131
28	1	0	-3.777164	1.534564	-0.449935
29	1	0	0.227031	0.951503	-0.645974
30	1	0	1.897327	-2.000180	-0.008911
31	8	0	2.135926	0.490489	-2.505436
32	1	0	2.156704	1.216958	-1.850455
33	1	0	1.471615	0.726996	-3.162648
34	8	0	-0.190159	3.553799	0.436067
35	1	0	-0.135537	3.826376	1.361802
36	1	0	-1.564279	2.089119	0.157133
37	8	0	2.416312	-2.163121	-1.846841
38	1	0	3.246819	-2.531150	-2.167659
39	1	0	2.391986	-1.227482	-2.136079
40	8	0	2.175866	2.391941	-0.501090
41	1	0	1.414628	2.914570	-0.183995
42	1	0	2.945327	2.972055	-0.465353
43	1	0	-0.468057	4.341849	-0.049812

(S,R)_2H2O_Blue_F

Energy: -654.180385 hartree

Center Number	Atomic Number	Atomic Type	Coordinates (Angstroms) X	Y	Z
1	6	0	2.096080	-0.260718	1.514456
2	6	0	1.019179	0.009885	-0.699078
3	6	0	2.341326	0.734344	-0.964150
4	6	0	3.499419	-0.201006	-0.607159
5	6	0	3.458199	-0.596745	0.888404
6	6	0	-0.216163	0.921575	-0.916470
7	6	0	-0.237911	2.176358	-0.041742
8	6	0	-1.661559	2.718094	0.092448
9	6	0	-2.563344	1.679609	0.740536
10	7	0	0.955791	-0.576540	0.652790
11	7	0	-2.389474	0.374493	0.080565
12	6	0	-1.394047	0.050517	-0.656369
13	1	0	2.056875	0.806797	1.760713
14	1	0	1.963999	-0.798306	2.457530
15	1	0	2.383576	1.027422	-2.018192
16	1	0	2.406418	1.655260	-0.372900

17	1	0	3.417209	-1.097847	-1.232632
18	1	0	4.455041	0.267742	-0.857683
19	1	0	3.651943	-1.669393	0.994590
20	1	0	4.242747	-0.075693	1.448346
21	1	0	0.942929	-0.806589	-1.430660
22	1	0	-0.261855	1.190025	-1.983536
23	1	0	0.149512	1.917766	0.951730
24	1	0	0.419054	2.937254	-0.468607
25	1	0	-1.679516	3.622501	0.704356
26	1	0	-2.058869	2.979879	-0.894766
27	1	0	-3.620154	1.936911	0.658205
28	1	0	-2.324116	1.537890	1.798864
29	1	0	-1.411119	-0.955274	-1.074775
30	1	0	0.821870	-1.581520	0.577101
31	8	0	-0.570920	-2.976628	-0.411342
32	1	0	-0.363042	-3.847993	-0.766880
33	1	0	-1.506950	-3.006455	-0.150917
34	8	0	-3.307186	-2.320902	0.155074
35	1	0	-3.925769	-2.633484	-0.518527
36	1	0	-3.090581	-0.358047	0.234044
37	1	0	-3.655644	-2.645753	0.995996

(S,R)_2H2O_Blue_R

Energy: -654.173855 hartree

Center Number	Atomic Number	Atomic Type	Coordinates (Angstroms) X	Y	Z
1	6	0	1.396501	-1.549986	1.190877
2	6	0	1.222845	-0.918620	-1.166050
3	6	0	2.484875	-0.131094	-1.156863
4	6	0	2.992553	0.139122	0.258618
5	6	0	2.855342	-1.124596	1.107630
6	6	0	-0.579897	1.668456	-1.424287
7	6	0	0.060689	2.778751	-0.627302
8	6	0	-0.639005	2.907137	0.730238
9	6	0	-0.735008	1.534632	1.399080
10	7	0	0.763102	-1.527579	-0.136291
11	7	0	-1.405732	0.559336	0.547654
12	6	0	-1.327768	0.722930	-0.820225
13	1	0	0.810595	-0.886375	1.834387
14	1	0	1.288839	-2.567455	1.570871

15	1	0	3.216086	-0.708302	-1.739862
16	1	0	2.306352	0.787986	-1.724070
17	1	0	4.031947	0.471010	0.219789
18	1	0	2.409653	0.950609	0.710172
19	1	0	3.446673	-1.933956	0.664579
20	1	0	3.228660	-0.961111	2.120874
21	1	0	0.654578	-1.028409	-2.085654
22	1	0	-0.516326	1.672579	-2.507700
23	1	0	1.136648	2.606126	-0.475994
24	1	0	-0.016751	3.722140	-1.178817
25	1	0	-0.099915	3.596016	1.386705
26	1	0	-1.649910	3.304950	0.582431
27	1	0	-1.266540	1.590466	2.351669
28	1	0	0.286661	1.187366	1.618071
29	1	0	-1.876881	-0.020129	-1.395403
30	1	0	-0.149690	-2.013638	-0.245259
31	8	0	-1.687389	-2.598439	-0.761732
32	1	0	-1.826512	-3.551163	-0.813964
33	1	0	-2.473497	-2.220755	-0.319547
34	8	0	-3.793092	-1.259376	0.462214
35	1	0	-4.508780	-0.972212	-0.119227
36	1	0	-2.254877	0.127633	0.890253
37	1	0	-4.221520	-1.673542	1.222020

(S,R)_2H2O_Blue_TS

Energy: -654.155727 hartree

Center Number	Atomic Number	Atomic Type	Coordinates (Angstroms) X	Y	Z
1	6	0	1.723810	-0.490805	1.480395
2	6	0	1.046055	-0.474645	-0.836152
3	6	0	2.389659	0.151432	-1.090601
4	6	0	3.439273	-0.742381	-0.399158
5	6	0	3.190873	-0.815082	1.120565
6	6	0	-0.258094	1.288900	-1.326964
7	6	0	0.113402	2.435073	-0.404787
8	6	0	-1.047905	2.764747	0.539513
9	6	0	-1.550896	1.492421	1.214550
10	7	0	0.834608	-0.900569	0.399879
11	7	0	-1.914174	0.506037	0.197547
12	6	0	-1.365740	0.526078	-1.018173

13	1	0	1.607923	0.584474	1.655878
14	1	0	1.409736	-1.002638	2.389865
15	1	0	2.561481	0.234231	-2.164881
16	1	0	2.449872	1.157810	-0.664232
17	1	0	3.387820	-1.743533	-0.838394
18	1	0	4.438377	-0.351414	-0.606169
19	1	0	3.444325	-1.812551	1.488221
20	1	0	3.831380	-0.102288	1.647660
21	1	0	0.574230	-1.058761	-1.618685
22	1	0	0.039479	1.329737	-2.370073
23	1	0	0.999393	2.202613	0.199036
24	1	0	0.376498	3.314189	-1.000510
25	1	0	-0.734788	3.479043	1.304979
26	1	0	-1.869641	3.216425	-0.027304
27	1	0	-2.433531	1.688654	1.827000
28	1	0	-0.772965	1.081093	1.870350
29	1	0	-1.775474	-0.189525	-1.726488
30	1	0	0.063869	-1.549279	0.551653
31	8	0	-1.271772	-2.612720	-0.389164
32	1	0	-1.152001	-3.560215	-0.519416
33	1	0	-2.218274	-2.477059	-0.204940
34	8	0	-3.876859	-1.715647	0.161334
35	1	0	-4.535254	-1.713423	-0.544861
36	1	0	-2.692957	-0.123744	0.366323
37	1	0	-4.336104	-2.029595	0.950521

(S,R)_3H2O_Blue_F

Energy: -730.569943 hartree

Center Number	Atomic Number	Atomic Type	Coordinates (Angstroms) X	Y	Z
1	6	0	1.643523	1.670649	-0.436507
2	6	0	1.521553	-0.487055	0.741819
3	6	0	2.014149	-0.978471	-0.627468
4	6	0	3.254202	-0.181935	-1.038532
5	6	0	2.887727	1.300758	-1.256612
6	6	0	0.078168	-0.973626	1.097171
7	6	0	-0.079712	-2.501554	1.146110
8	6	0	-0.623790	-3.070432	-0.166383
9	6	0	-1.945673	-2.410553	-0.523495
10	7	0	1.679616	0.964494	0.842071

11	7	0	-1.817635	-0.944965	-0.450408
12	6	0	-0.931845	-0.316444	0.233649
13	1	0	0.737041	1.443573	-1.026293
14	1	0	1.616690	2.744413	-0.231883
15	1	0	2.235474	-2.048583	-0.571987
16	1	0	1.234841	-0.852867	-1.392083
17	1	0	4.002900	-0.271041	-0.243281
18	1	0	3.698257	-0.607285	-1.942767
19	1	0	3.727654	1.937848	-0.962756
20	1	0	2.687709	1.493504	-2.316046
21	1	0	2.155813	-0.953110	1.508806
22	1	0	-0.122457	-0.543866	2.089712
23	1	0	0.886189	-2.955913	1.387244
24	1	0	-0.765502	-2.766335	1.957269
25	1	0	0.086586	-2.913919	-0.983789
26	1	0	-0.786207	-4.146750	-0.077043
27	1	0	-2.745183	-2.700027	0.165352
28	1	0	-2.265268	-2.647879	-1.538825
29	1	0	-0.966533	0.771682	0.176148
30	1	0	1.025762	1.364195	1.512082
31	8	0	-1.663054	2.905373	-0.071762
32	1	0	-2.322876	2.495803	-0.659144
33	1	0	-1.144601	3.513099	-0.611214
34	8	0	-3.357894	1.198622	-1.558677
35	1	0	-4.289892	1.229357	-1.303886
36	1	0	-2.490471	-0.368390	-0.973575
37	8	0	-0.812883	1.915489	2.374000
38	1	0	-0.923045	2.418047	3.188719
39	1	0	-1.200920	2.451697	1.659171
40	1	0	-3.344986	1.312142	-2.518582

(S,R)_3H2O_Blue_R

Energy: -730.566319 hartree

Center Number	Atomic Number	Atomic Type	Coordinates (Angstroms) X	Y	Z
1	6	0	-2.387580	1.013804	-0.208228
2	6	0	-0.444964	1.726641	1.101487
3	6	0	0.157239	2.484899	-0.022733
4	6	0	-0.807239	2.680929	-1.191923
5	6	0	-1.572655	1.380841	-1.438860

6	6	0	1.603778	-0.790425	1.379443
7	6	0	3.040603	-0.337093	1.478728
8	6	0	3.529489	0.239672	0.142297
9	6	0	3.017188	-0.592458	-1.032968
10	7	0	-1.565042	1.110174	1.011947
11	7	0	1.557206	-0.591331	-1.024633
12	6	0	0.972083	-0.901868	0.197262
13	1	0	-2.754466	-0.015478	-0.245737
14	1	0	-3.243480	1.682193	-0.073647
15	1	0	0.555366	3.425327	0.369548
16	1	0	1.024951	1.882674	-0.335158
17	1	0	-1.510573	3.489426	-0.964061
18	1	0	-0.245914	2.976102	-2.080710
19	1	0	-2.249578	1.472547	-2.291176
20	1	0	-0.861205	0.575319	-1.658766
21	1	0	0.074381	1.658921	2.053937
22	1	0	1.056957	-1.029647	2.286834
23	1	0	3.143944	0.418998	2.266211
24	1	0	3.680185	-1.176776	1.781736
25	1	0	3.167318	1.267769	0.026256
26	1	0	4.622200	0.279075	0.117069
27	1	0	3.406488	-1.619525	-0.944215
28	1	0	3.363130	-0.179080	-1.983370
29	1	0	-0.069988	-1.211423	0.139022
30	1	0	-1.876692	0.515911	1.798017
31	8	0	-2.391987	-2.414703	-0.010850
32	1	0	-1.848944	-2.365418	-0.821936
33	1	0	-3.137249	-2.992253	-0.211310
34	8	0	-0.774316	-2.109432	-2.273330
35	1	0	-0.438304	-2.954887	-2.599300
36	1	0	1.111240	-1.056485	-1.807080
37	8	0	-2.354640	-1.118843	2.420489
38	1	0	-1.837395	-1.529703	3.122351
39	1	0	-2.310148	-1.714074	1.645827
40	1	0	-1.247473	-1.717253	-3.019054

(S,R)_3H2O_Blue_TS

Energy: -730.545289 hartree

Center Number	Atomic Number	Atomic Type	Coordinates (Angstroms) X	Y	Z

1	6	0	-2.004245	1.404398	-0.236334
2	6	0	0.294750	1.714319	0.649972
3	6	0	0.855235	2.016994	-0.725758
4	6	0	-0.253323	2.436701	-1.693608
5	6	0	-1.362431	1.389994	-1.623159
6	6	0	1.070550	-0.162069	1.320916
7	6	0	2.572246	-0.064393	1.470271
8	6	0	3.304805	-0.302608	0.140535
9	6	0	2.722116	-1.488960	-0.626743
10	7	0	-1.033148	1.742707	0.807806
11	7	0	1.264277	-1.379491	-0.727756
12	6	0	0.539740	-0.905402	0.283698
13	1	0	-2.457409	0.434008	0.000111
14	1	0	-2.795751	2.159380	-0.194585
15	1	0	1.622424	2.789191	-0.619586
16	1	0	1.346984	1.131812	-1.141584
17	1	0	-0.647585	3.422819	-1.422444
18	1	0	0.153725	2.508157	-2.704979
19	1	0	-2.137751	1.566468	-2.372449
20	1	0	-0.928771	0.403839	-1.834057
21	1	0	0.847416	2.101483	1.501068
22	1	0	0.451851	-0.082541	2.208743
23	1	0	2.853300	0.916904	1.872465
24	1	0	2.905229	-0.800807	2.212016
25	1	0	3.254697	0.592241	-0.485287
26	1	0	4.364553	-0.495549	0.326569
27	1	0	2.967641	-2.428151	-0.115877
28	1	0	3.129825	-1.537398	-1.637551
29	1	0	-0.535709	-1.055979	0.206062
30	1	0	-1.382006	1.609459	1.751699
31	8	0	-2.804358	-2.015420	0.443700
32	1	0	-2.343469	-2.243167	-0.385579
33	1	0	-3.746974	-2.016173	0.241068
34	8	0	-1.222801	-2.510791	-1.839442
35	1	0	-1.146856	-3.458573	-2.013870
36	1	0	0.766955	-1.883556	-1.454259
37	8	0	-2.035536	-0.301253	2.534837
38	1	0	-2.189050	-0.694467	3.400870
39	1	0	-2.318544	-0.965582	1.879139
40	1	0	-1.587716	-2.130462	-2.649546

(S,R)_4H2O_Blue_F

Energy: -806.954985 hartree

Center Number	Atomic Number	Atomic Type	Coordinates (Angstroms) X	Y	Z
1	6	0	1.728918	0.595401	-0.455892
2	6	0	-0.244066	1.576743	-1.352012
3	6	0	-0.066054	2.653246	-0.348235
4	6	0	1.411823	3.097713	-0.194481
5	6	0	2.412452	1.962215	-0.485816
6	6	0	-2.718567	-1.140790	-0.515718
7	6	0	-3.837804	-0.203470	-0.130268
8	6	0	-3.316681	0.916397	0.779359
9	6	0	-2.349381	0.361475	1.824627
10	7	0	0.598878	0.614800	-1.388763
11	7	0	-1.208586	-0.250688	1.150264
12	6	0	-1.531113	-1.124599	0.113248
13	1	0	1.324092	0.375898	0.538418
14	1	0	2.395765	-0.215505	-0.749535
15	1	0	-0.729604	3.491005	-0.561392
16	1	0	-0.403634	2.191630	0.593796
17	1	0	1.598142	3.935033	-0.869710
18	1	0	1.538957	3.470717	0.823570
19	1	0	2.858441	2.094486	-1.475638
20	1	0	3.223176	1.951825	0.244748
21	1	0	-1.083368	1.541714	-2.042678
22	1	0	-2.863300	-1.835598	-1.337035
23	1	0	-4.292590	0.231843	-1.027770
24	1	0	-4.640315	-0.755011	0.377750
25	1	0	-2.789447	1.665083	0.175197
26	1	0	-4.144267	1.429572	1.277301
27	1	0	-2.880192	-0.375513	2.448790
28	1	0	-1.984729	1.157527	2.480193
29	1	0	-0.722628	-1.786999	-0.188842
30	1	0	0.478651	-0.201028	-2.028073
31	8	0	1.294552	-1.858196	1.783049
32	1	0	2.118875	-1.340093	1.900665
33	1	0	1.223858	-2.434627	2.554051
34	8	0	3.533843	-0.258190	1.861243
35	1	0	3.683406	0.297201	2.636362
36	1	0	-0.486441	-0.619808	1.760606
37	8	0	1.229730	-3.281083	-0.572483
38	1	0	1.996451	-3.861112	-0.643707
39	1	0	1.317521	-2.801270	0.279352
40	8	0	0.487542	-1.770781	-2.706884

41	1	0	1.040661	-1.913901	-3.483586
42	1	0	0.803876	-2.389413	-2.009991
43	1	0	4.384102	-0.672432	1.668393

(S,R)_4H2O_Blue_R

Energy: -806.960299 hartree

Center Number	Atomic Number	Atomic Type	Coordinates (Angstroms) X	Y	Z
1	6	0	2.238634	0.137608	-1.135749
2	6	0	-0.039167	0.963749	-1.475403
3	6	0	0.274881	2.324167	-0.963076
4	6	0	1.577849	2.340411	-0.166822
5	6	0	2.657114	1.586289	-0.942903
6	6	0	-3.044319	-1.070095	-0.108752
7	6	0	-4.046879	0.052194	0.011715
8	6	0	-3.344287	1.379780	0.324375
9	6	0	-2.248592	1.177326	1.370325
10	7	0	0.839764	0.036189	-1.584454
11	7	0	-1.256231	0.240187	0.853454
12	6	0	-1.774292	-0.936286	0.307947
13	1	0	2.311869	-0.418243	-0.194500
14	1	0	2.847126	-0.374735	-1.883425
15	1	0	0.339823	2.981230	-1.842222
16	1	0	-0.582104	2.672661	-0.381258
17	1	0	1.881818	3.371135	0.026775
18	1	0	1.415850	1.853521	0.803301
19	1	0	2.812742	2.060063	-1.918985
20	1	0	3.606900	1.597089	-0.405193
21	1	0	-1.037498	0.727242	-1.836173
22	1	0	-3.350549	-2.011845	-0.553031
23	1	0	-4.620966	0.146755	-0.916994
24	1	0	-4.779018	-0.177143	0.797521
25	1	0	-2.890172	1.779864	-0.590957
26	1	0	-4.062046	2.124562	0.679227
27	1	0	-2.701821	0.796055	2.299292
28	1	0	-1.749697	2.122306	1.606370
29	1	0	-1.053756	-1.744269	0.199999
30	1	0	0.548560	-0.900554	-1.929675
31	8	0	1.136154	-1.083616	2.098178
32	1	0	1.981194	-0.586782	2.137882

33	1	0	0.929865	-1.337882	3.006415
34	8	0	3.464972	0.351533	1.943646
35	1	0	3.545010	1.159819	2.465535
36	1	0	-0.461623	0.068165	1.463255
37	8	0	0.928378	-3.286245	0.443318
38	1	0	1.643584	-3.911988	0.605647
39	1	0	1.081314	-2.524738	1.041614
40	8	0	0.245717	-2.601588	-2.102719
41	1	0	0.726071	-3.077859	-2.789646
42	1	0	0.533591	-2.971775	-1.238832
43	1	0	4.291792	-0.128822	2.075719

(S,R)_4H2O_Blue_TS

Energy: -806.950977 hartree

Center Number	Atomic Number	Atomic Type	Coordinates (Angstroms) X	Y	Z
1	6	0	1.741058	0.503303	-0.562174
2	6	0	-0.229398	1.548068	-1.402828
3	6	0	-0.001012	2.640368	-0.426690
4	6	0	1.494363	2.989767	-0.202878
5	6	0	2.446683	1.855357	-0.614939
6	6	0	-2.762815	-1.113285	-0.470790
7	6	0	-3.855864	-0.146838	-0.081500
8	6	0	-3.295529	0.982222	0.793073
9	6	0	-2.311610	0.431422	1.824692
10	7	0	0.574545	0.553831	-1.448067
11	7	0	-1.196243	-0.209661	1.134865
12	6	0	-1.559740	-1.102925	0.127959
13	1	0	1.374659	0.287007	0.447538
14	1	0	2.375536	-0.320857	-0.890790
15	1	0	-0.599071	3.512222	-0.692135
16	1	0	-0.419279	2.230978	0.507278
17	1	0	1.735443	3.893057	-0.766595
18	1	0	1.626249	3.225725	0.855206
19	1	0	2.805810	2.009653	-1.637109
20	1	0	3.315854	1.817597	0.044111
21	1	0	-1.094425	1.527522	-2.061805
22	1	0	-2.939905	-1.826338	-1.269884
23	1	0	-4.321742	0.277865	-0.978555
24	1	0	-4.657253	-0.672992	0.454513

25	1	0	-2.772502	1.710879	0.161065
26	1	0	-4.103176	1.517421	1.300595
27	1	0	-2.838629	-0.286737	2.473801
28	1	0	-1.920183	1.232958	2.457999
29	1	0	-0.770982	-1.787695	-0.177829
30	1	0	0.400143	-0.268925	-2.063024
31	8	0	1.290208	-1.807524	1.851099
32	1	0	2.103642	-1.275059	1.980291
33	1	0	1.203030	-2.368437	2.631667
34	8	0	3.473677	-0.138706	1.934521
35	1	0	3.615837	0.404421	2.719604
36	1	0	-0.464978	-0.575872	1.736170
37	8	0	1.277013	-3.251347	-0.493307
38	1	0	2.092923	-3.758131	-0.575760
39	1	0	1.338083	-2.758191	0.353049
40	8	0	0.373232	-1.883861	-2.668219
41	1	0	0.879831	-2.068159	-3.467606
42	1	0	0.739753	-2.455427	-1.956515
43	1	0	4.337365	-0.506927	1.710827

(S,R)_4H2O_Orange_F

Energy: -806.959933 hartree

Center Number	Atomic Number	Atomic Type	Coordinates (Angstroms) X	Y	Z
1	6	0	-1.363490	-1.346943	0.836327
2	6	0	0.484317	-1.472235	-0.784740
3	6	0	1.177202	-2.109739	0.429646
4	6	0	0.305498	-3.239965	0.980910
5	6	0	-1.012487	-2.666437	1.540015
6	6	0	1.088487	-0.083459	-1.191370
7	6	0	2.587362	-0.113632	-1.526655
8	6	0	3.461215	0.241253	-0.320293
9	6	0	3.066045	1.593581	0.251835
10	7	0	-0.960447	-1.430802	-0.565264
11	7	0	1.607130	1.663229	0.441836
12	6	0	0.746154	0.940979	-0.177805
13	1	0	-0.877036	-0.509725	1.371186
14	1	0	-2.437561	-1.152234	0.866472
15	1	0	2.162851	-2.480660	0.133227
16	1	0	1.340053	-1.365896	1.221909

17	1	0	0.095192	-3.945511	0.169314
18	1	0	0.844960	-3.794534	1.753766
19	1	0	-1.822121	-3.389016	1.397408
20	1	0	-0.923528	-2.485565	2.616665
21	1	0	0.680778	-2.112805	-1.655603
22	1	0	0.510841	0.215911	-2.078020
23	1	0	2.851834	-1.103985	-1.909845
24	1	0	2.787762	0.601385	-2.331165
25	1	0	3.373387	-0.517907	0.461987
26	1	0	4.512420	0.285127	-0.613537
27	1	0	3.340869	2.415073	-0.416699
28	1	0	3.520882	1.777654	1.225580
29	1	0	-0.300326	1.130922	0.065059
30	1	0	-1.412220	-0.730085	-1.147591
31	8	0	-4.063211	0.083083	-0.497579
32	1	0	-5.010280	0.170010	-0.653880
33	1	0	-3.624144	0.281323	-1.343110
34	8	0	-0.291303	2.939676	2.146549
35	1	0	-0.314865	3.903153	2.220756
36	1	0	1.225509	2.334969	1.119287
37	8	0	-2.027867	1.017658	-2.194731
38	1	0	-1.973057	1.724075	-1.526352
39	1	0	-2.010485	1.436003	-3.063020
40	8	0	-2.267899	2.064659	0.304992
41	1	0	-2.979756	1.428251	0.485060
42	1	0	-1.858755	2.345274	1.138739
43	1	0	-0.269561	2.610772	3.055333

(S,R)_4H2O_Orange_R

Energy: -806.950880 hartree

Center Number	Atomic Number	Atomic Type	Coordinates (Angstroms) X	Y	Z
1	6	0	-1.559853	-1.400412	1.091146
2	6	0	-0.602887	-1.130490	-1.073778
3	6	0	0.427413	-2.090708	-0.615094
4	6	0	-0.231143	-3.318397	0.062673
5	6	0	-1.405341	-2.922327	0.985615
6	6	0	1.839554	1.074438	-1.316710
7	6	0	3.222596	0.505131	-1.525080
8	6	0	3.702181	-0.265282	-0.285260

9	6	0	3.287118	0.448422	1.001127
10	7	0	-1.546157	-0.824914	-0.257511
11	7	0	1.832666	0.569945	1.040474
12	6	0	1.250989	1.096444	-0.107282
13	1	0	-0.723574	-0.956713	1.643640
14	1	0	-2.493146	-1.109627	1.571669
15	1	0	1.082504	-2.374010	-1.439048
16	1	0	1.032230	-1.552018	0.131513
17	1	0	-0.583757	-3.999854	-0.714148
18	1	0	0.544786	-3.842840	0.623114
19	1	0	-2.342641	-3.331235	0.600216
20	1	0	-1.267073	-3.318938	1.993443
21	1	0	-0.627414	-0.670546	-2.057495
22	1	0	1.296741	1.477239	-2.166509
23	1	0	3.228542	-0.160803	-2.396433
24	1	0	3.927953	1.313365	-1.760327
25	1	0	3.269030	-1.271548	-0.280593
26	1	0	4.789379	-0.383774	-0.304787
27	1	0	3.760300	1.443198	1.030877
28	1	0	3.617268	-0.110699	1.879918
29	1	0	0.245803	1.487843	0.030598
30	1	0	-2.312177	-0.197499	-0.539648
31	8	0	-3.892482	0.780717	-0.721399
32	1	0	-4.775048	0.408572	-0.832669
33	1	0	-3.615830	1.150814	-1.576529
34	8	0	-0.330795	1.847980	2.749434
35	1	0	-0.066977	2.671517	3.180331
36	1	0	1.441989	0.949636	1.895643
37	8	0	-1.704951	1.555554	-2.104084
38	1	0	-1.549960	2.056934	-1.280484
39	1	0	-1.357644	2.084277	-2.830818
40	8	0	-1.957651	2.388941	0.509686
41	1	0	-2.825369	1.960315	0.556645
42	1	0	-1.483515	2.189814	1.337887
43	1	0	-0.714695	1.304905	3.450027

(S,R)_4H2O_Orange_TS

Energy: -806.937301 hartree

Center Number	Atomic Number	Atomic Type	Coordinates (Angstroms) X	Y	Z

1	6	0	-1.472090	-1.163615	0.844703
2	6	0	0.085705	-1.281328	-0.976864
3	6	0	0.931175	-2.079709	-0.023383
4	6	0	0.046656	-3.189859	0.574827
5	6	0	-1.132196	-2.580142	1.354174
6	6	0	1.247216	0.584367	-1.297413
7	6	0	2.661911	0.224118	-1.689197
8	6	0	3.571423	0.023449	-0.465151
9	6	0	3.326870	1.087806	0.603502
10	7	0	-1.155758	-1.045447	-0.571225
11	7	0	1.903658	1.157059	0.929655
12	6	0	0.986373	1.085000	-0.041067
13	1	0	-0.885610	-0.415306	1.397387
14	1	0	-2.527676	-0.925222	0.970989
15	1	0	1.797036	-2.488103	-0.546783
16	1	0	1.296737	-1.441862	0.792954
17	1	0	-0.322732	-3.820938	-0.239210
18	1	0	0.653149	-3.824570	1.224936
19	1	0	-2.010782	-3.223632	1.262707
20	1	0	-0.890881	-2.507171	2.418454
21	1	0	0.225716	-1.402155	-2.046489
22	1	0	0.528560	0.824835	-2.073613
23	1	0	2.667706	-0.685560	-2.304141
24	1	0	3.072715	1.017150	-2.326745
25	1	0	3.403681	-0.960916	-0.020100
26	1	0	4.621128	0.060002	-0.768968
27	1	0	3.659709	2.069907	0.242784
28	1	0	3.878425	0.857103	1.516234
29	1	0	-0.021571	1.383982	0.237971
30	1	0	-1.831476	-0.613391	-1.193102
31	8	0	-3.895525	0.195211	-0.771955
32	1	0	-4.819014	-0.062856	-0.868368
33	1	0	-3.639399	0.667084	-1.582214
34	8	0	-0.407360	1.924043	2.663696
35	1	0	-0.318572	2.802300	3.057289
36	1	0	1.596731	1.495963	1.833413
37	8	0	-1.906806	1.445597	-2.142182
38	1	0	-1.814619	1.930731	-1.300187
39	1	0	-1.742297	2.073085	-2.854857
40	8	0	-2.284042	2.106550	0.514159
41	1	0	-2.984946	1.434564	0.490308
42	1	0	-1.798684	2.008758	1.351362
43	1	0	-0.596724	1.333203	3.404431

(S,R)_4H2O_Pink_F

Energy: -806.962519 hartree

Center Number	Atomic Number	Atomic Type	Coordinates (Angstroms) X	Y	Z
1	6	0	-2.354444	0.796565	-0.050686
2	6	0	-1.486236	-1.475001	-0.515953
3	6	0	-1.795200	-1.617866	0.982189
4	6	0	-3.235230	-1.180253	1.256460
5	6	0	-3.421641	0.317630	0.939793
6	6	0	0.031109	-1.577005	-0.837461
7	6	0	0.688787	-2.888159	-0.400262
8	6	0	2.209193	-2.780198	-0.520862
9	6	0	2.724566	-1.675582	0.389595
10	7	0	-2.100239	-0.248579	-1.041779
11	7	0	1.929756	-0.448699	0.208128
12	6	0	0.768629	-0.390488	-0.329371
13	1	0	-1.447642	1.096035	0.503319
14	1	0	-2.691550	1.688450	-0.584343
15	1	0	-1.650181	-2.657695	1.285206
16	1	0	-1.100148	-1.011697	1.581552
17	1	0	-3.900344	-1.782204	0.626484
18	1	0	-3.504800	-1.393662	2.294508
19	1	0	-4.417884	0.487309	0.519667
20	1	0	-3.348901	0.915942	1.854162
21	1	0	-1.941507	-2.332746	-1.030729
22	1	0	0.108371	-1.485922	-1.934430
23	1	0	0.429234	-3.105909	0.641608
24	1	0	0.303519	-3.708312	-1.011248
25	1	0	2.692112	-3.717829	-0.238018
26	1	0	2.491292	-2.561879	-1.556767
27	1	0	3.760635	-1.407355	0.176995
28	1	0	2.653149	-1.952567	1.445832
29	1	0	0.346469	0.606296	-0.432311
30	1	0	-1.579964	0.128622	-1.829135
31	8	0	-0.815710	3.772634	0.463992
32	1	0	-0.938319	4.726152	0.393015
33	1	0	-0.954210	3.410484	-0.429721
34	8	0	3.977976	1.430205	1.108241
35	1	0	4.795463	1.416887	0.595075
36	1	0	2.365259	0.430826	0.512715
37	8	0	-0.273952	2.298022	-1.883933

38	1	0	-0.363107	2.548629	-2.811105
39	1	0	0.654973	2.451168	-1.639980
40	8	0	1.609365	2.476197	0.104708
41	1	0	0.954875	3.068248	0.518693
42	1	0	2.469751	2.621369	0.525175
43	1	0	4.248204	1.407980	2.034626

(S,R)_4H2O_Pink_R

Energy: -806.945735 hartree

Center Number	Atomic Number	Atomic Type	Coordinates (Angstroms) X	Y	Z
1	6	0	-2.090746	-1.440775	-0.454259
2	6	0	0.010289	-2.219509	0.379682
3	6	0	-0.122839	-1.178623	1.425720
4	6	0	-1.548641	-1.165935	2.029506
5	6	0	-2.651184	-1.377548	0.969840
6	6	0	2.168459	-0.918975	-1.414002
7	6	0	3.502705	-1.130501	-0.738199
8	6	0	4.001857	0.180639	-0.118117
9	6	0	2.865361	0.891088	0.614962
10	7	0	-0.937883	-2.352182	-0.478286
11	7	0	1.799503	1.201265	-0.328656
12	6	0	1.429036	0.186689	-1.196093
13	1	0	-1.731940	-0.457741	-0.776654
14	1	0	-2.825669	-1.803567	-1.170169
15	1	0	0.645644	-1.311296	2.187362
16	1	0	0.044712	-0.210570	0.932569
17	1	0	-1.611281	-1.946469	2.790674
18	1	0	-1.675932	-0.207280	2.536374
19	1	0	-3.184121	-2.311563	1.166469
20	1	0	-3.382769	-0.567600	0.986807
21	1	0	0.850314	-2.904964	0.321681
22	1	0	1.814290	-1.646324	-2.138300
23	1	0	3.427665	-1.905311	0.038316
24	1	0	4.236126	-1.501853	-1.462983
25	1	0	4.827869	-0.006012	0.573888
26	1	0	4.373977	0.842844	-0.908454
27	1	0	3.208513	1.822172	1.072148
28	1	0	2.496352	0.236377	1.422612
29	1	0	0.488666	0.350645	-1.719818

30	1	0	-0.874486	-3.068058	-1.197289
31	8	0	-3.855966	1.123713	-0.789446
32	1	0	-4.678517	1.621564	-0.854440
33	1	0	-3.363449	1.289167	-1.614452
34	8	0	0.486199	2.638197	2.077578
35	1	0	0.608944	3.555956	2.348999
36	1	0	1.044264	1.758722	0.052173
37	8	0	-1.660872	1.512584	-2.455837
38	1	0	-1.489727	2.171211	-3.138324
39	1	0	-1.273092	1.851166	-1.627982
40	8	0	-1.325231	1.670161	0.249016
41	1	0	-2.295560	1.649489	0.311213
42	1	0	-0.964206	2.135654	1.022854
43	1	0	0.713245	2.103526	2.848586

(S,R)_4H2O_Pink_TS

Energy: -806.935645 hartree

Center Number	Atomic Number	Atomic Type	Coordinates (Angstroms) X	Y	Z
1	6	0	-1.934522	-1.475636	-0.359742
2	6	0	0.349369	-2.138895	-0.057102
3	6	0	0.253377	-1.415589	1.254128
4	6	0	-1.010188	-1.934080	1.970753
5	6	0	-2.267801	-1.624965	1.138727
6	6	0	1.830410	-0.879274	-1.309765
7	6	0	3.183016	-1.143485	-0.679873
8	6	0	3.814176	0.166682	-0.193732
9	6	0	2.808621	0.972727	0.624406
10	7	0	-0.784287	-2.306029	-0.706795
11	7	0	1.601527	1.201330	-0.160229
12	6	0	1.196799	0.320824	-1.085822
13	1	0	-1.672092	-0.434124	-0.578268
14	1	0	-2.771665	-1.755637	-0.997420
15	1	0	1.158863	-1.589760	1.838534
16	1	0	0.142565	-0.334749	1.099150
17	1	0	-0.911694	-3.012815	2.127018
18	1	0	-1.078318	-1.470062	2.957459
19	1	0	-3.008930	-2.416567	1.276036
20	1	0	-2.725625	-0.686172	1.460576
21	1	0	1.121824	-2.882091	-0.224794

22	1	0	1.535595	-1.440577	-2.189280
23	1	0	3.102442	-1.843024	0.165909
24	1	0	3.841605	-1.625315	-1.408839
25	1	0	4.699934	-0.030380	0.415402
26	1	0	4.126954	0.764188	-1.057317
27	1	0	3.216124	1.944790	0.911972
28	1	0	2.557445	0.429081	1.545883
29	1	0	0.288868	0.585630	-1.624232
30	1	0	-0.826373	-2.922772	-1.507779
31	8	0	-3.951408	0.908949	-0.258141
32	1	0	-4.817042	1.330397	-0.217506
33	1	0	-3.599989	1.083854	-1.149943
34	8	0	0.489476	3.523367	1.394782
35	1	0	0.500398	4.453458	1.137507
36	1	0	1.028953	2.005140	0.069042
37	8	0	-2.028471	1.476329	-2.227892
38	1	0	-2.026566	2.191580	-2.873818
39	1	0	-1.605892	1.821667	-1.417571
40	8	0	-1.367583	1.708245	0.421957
41	1	0	-2.316520	1.611607	0.611598
42	1	0	-1.011321	2.438127	0.954502
43	1	0	0.854120	3.492438	2.287826

Nitramidine_conformationA_F.log
Energy: -939.983275 hartree

Center Number	Atomic Number	Atomic Type	Coordinates (Angstroms) X	Y	Z
1	6	0	3.257383	-0.992921	0.267949
2	6	0	3.378604	0.313708	-0.284290
3	6	0	4.638073	0.764932	-0.737274
4	6	0	5.721526	-0.079196	-0.617117
5	6	0	5.582220	-1.373426	-0.051095
6	6	0	4.365492	-1.847591	0.398096
7	6	0	1.243407	-0.051431	0.316926
8	6	0	2.084183	0.898834	-0.233463
9	7	0	1.948451	-1.202523	0.605740
10	6	0	1.521720	2.187847	-0.746354
11	6	0	0.252139	2.531708	0.031421
12	6	0	-0.165628	0.154442	0.404417
13	6	0	-2.089942	1.510121	0.380649
14	6	0	-1.227927	-0.870963	0.619099

15	6	0	-2.820907	0.625587	-0.661205
16	6	0	-2.103217	-0.756789	-0.684827
17	7	0	-0.632654	1.360474	0.182824
18	6	0	-4.332176	0.588109	-0.374639
19	6	0	-4.378580	-1.869267	-0.570219
20	6	0	-5.014480	-0.582972	-1.073456
21	7	0	-2.972547	-1.884265	-0.960079
22	6	0	-2.056412	-0.487041	1.870115
23	6	0	-2.347742	1.025199	1.822025
24	1	0	4.745199	1.756802	-1.165152
25	1	0	6.699811	0.244806	-0.955742
26	1	0	6.458865	-2.008067	0.029471
27	1	0	4.263820	-2.837946	0.828363
28	1	0	1.593512	-2.018130	1.084656
29	1	0	2.229319	3.012118	-0.631128
30	1	0	1.303390	2.092883	-1.817487
31	1	0	0.500986	2.875966	1.043172
32	1	0	-0.320376	3.309967	-0.472333
33	1	0	-2.338942	2.564114	0.252309
34	1	0	-0.802310	-1.873194	0.696791
35	1	0	-2.685690	1.083424	-1.646217
36	1	0	-1.378958	-0.726254	-1.507453
37	1	0	-4.766646	1.546758	-0.676104
38	1	0	-4.526525	0.476542	0.696743
39	1	0	-4.848172	-2.743250	-1.030494
40	1	0	-4.543623	-1.946389	0.517908
41	1	0	-6.085182	-0.578882	-0.849502
42	1	0	-4.894162	-0.517161	-2.161467
43	1	0	-2.517636	-2.764939	-0.748167
44	1	0	-2.977296	-1.075789	1.850717
45	1	0	-1.516876	-0.756762	2.779747
46	1	0	-3.365633	1.259553	2.133812
47	1	0	-1.676026	1.574674	2.487610

Nitramidine_conformationA_R.log

Energy: -939.973640 hartree

Center Number	Atomic Number	Atomic Type	Coordinates (Angstroms) X	Y	Z
1	6	0	3.499069	-0.894900	0.135675
2	6	0	3.375382	0.419023	-0.389798

3	6	0	4.493204	1.026072	-0.989969
4	6	0	5.685559	0.321081	-1.050527
5	6	0	5.786936	-0.984206	-0.521126
6	6	0	4.702058	-1.609052	0.077150
7	6	0	1.396080	-0.210178	0.487078
8	6	0	2.023548	0.832547	-0.147121
9	7	0	2.280502	-1.254575	0.662251
10	6	0	1.297498	2.105790	-0.450232
11	6	0	0.115236	2.230817	0.510475
12	6	0	-0.008866	-0.202682	0.895455
13	6	0	-2.081123	1.085729	0.896017
14	6	0	-0.635780	-1.263909	1.453466
15	6	0	-2.940848	0.698342	-0.350752
16	6	0	-2.417607	-0.497259	-1.063277
17	7	0	-0.666427	0.988204	0.554318
18	6	0	-4.447445	0.605557	-0.103173
19	6	0	-4.654016	-1.319666	-1.675425
20	6	0	-5.149672	0.081516	-1.355378
21	7	0	-3.177279	-1.361304	-1.635437
22	6	0	-2.038680	-1.177744	1.988839
23	6	0	-2.411678	0.290465	2.162271
24	1	0	4.420923	2.030045	-1.398652
25	1	0	6.557269	0.775215	-1.510886
26	1	0	6.734341	-1.510328	-0.583320
27	1	0	4.781231	-2.612291	0.483911
28	1	0	2.085447	-2.128012	1.127761
29	1	0	1.953388	2.973763	-0.327117
30	1	0	0.936940	2.113852	-1.486969
31	1	0	0.480792	2.490163	1.516250
32	1	0	-0.554694	3.026354	0.177687
33	1	0	-2.306312	2.138271	1.088690
34	1	0	-0.091931	-2.195371	1.569749
35	1	0	-2.743584	1.507641	-1.074565
36	1	0	-1.345065	-0.642472	-1.160145
37	1	0	-4.828311	1.589910	0.180708
38	1	0	-4.652899	-0.076372	0.729059
39	1	0	-4.947957	-1.646468	-2.673457
40	1	0	-5.014045	-2.055187	-0.950010
41	1	0	-6.230606	0.038617	-1.208044
42	1	0	-4.956971	0.744434	-2.205534
43	1	0	-2.730812	-2.140696	-2.114435
44	1	0	-2.760594	-1.691547	1.335034
45	1	0	-2.098553	-1.697226	2.950919
46	1	0	-3.462326	0.416663	2.432126
47	1	0	-1.816367	0.720703	2.975430

Nitramidine_conformationA_TS.log

Energy: -939.960630 hartree

Center Number	Atomic Number	Atomic Type	Coordinates (Angstroms) X	Y	Z
1	6	0	3.243927	-0.821410	0.504294
2	6	0	3.229501	0.266321	-0.411099
3	6	0	4.392840	0.564199	-1.147366
4	6	0	5.520784	-0.212482	-0.951724
5	6	0	5.515961	-1.287081	-0.031150
6	6	0	4.388444	-1.606248	0.706654
7	6	0	1.212832	0.099662	0.571657
8	6	0	1.921407	0.841514	-0.344033
9	7	0	2.002070	-0.908391	1.083060
10	6	0	1.273899	1.981692	-1.070037
11	6	0	0.147483	2.536643	-0.195690
12	6	0	-0.190323	0.326351	0.864332
13	6	0	-2.152807	1.529333	0.326252
14	6	0	-1.056528	-0.630091	1.376159
15	6	0	-2.680322	0.495352	-0.725666
16	6	0	-1.908797	-0.808180	-0.690103
17	7	0	-0.703244	1.466125	0.339129
18	6	0	-4.206819	0.316519	-0.663494
19	6	0	-3.998344	-2.148141	-0.819624
20	6	0	-4.656760	-0.922143	-1.433184
21	7	0	-2.542682	-1.964904	-0.828745
22	6	0	-2.342984	-0.235944	2.067901
23	6	0	-2.663536	1.224536	1.743199
24	1	0	4.400600	1.389614	-1.853152
25	1	0	6.426630	0.001596	-1.509482
26	1	0	6.418719	-1.875302	0.099570
27	1	0	4.386901	-2.431158	1.411603
28	1	0	1.741999	-1.547421	1.820334
29	1	0	1.990724	2.782633	-1.272721
30	1	0	0.876905	1.645634	-2.036762
31	1	0	0.574652	3.102827	0.641530
32	1	0	-0.495298	3.206561	-0.768463
33	1	0	-2.455493	2.529618	0.011965
34	1	0	-0.639889	-1.607469	1.602775
35	1	0	-2.427695	0.923981	-1.704842

36	1	0	-0.901021	-0.795897	-1.093233
37	1	0	-4.674079	1.220929	-1.063768
38	1	0	-4.548265	0.212858	0.370262
39	1	0	-4.205192	-3.050330	-1.396802
40	1	0	-4.356165	-2.312826	0.202962
41	1	0	-5.742550	-1.030759	-1.380316
42	1	0	-4.376267	-0.847566	-2.490074
43	1	0	-1.974747	-2.788318	-0.983119
44	1	0	-3.154150	-0.906218	1.759191
45	1	0	-2.235997	-0.370534	3.149363
46	1	0	-3.728930	1.442239	1.837315
47	1	0	-2.133548	1.893094	2.428092

Nitramidine_conformationB_F.log
Energy: -939.983123 hartree

Center Number	Atomic Number	Atomic Type	Coordinates (Angstroms) X	Y	Z
1	6	0	3.233710	-1.078840	0.050466
2	6	0	3.429850	0.319957	-0.127763
3	6	0	4.741255	0.829305	-0.253670
4	6	0	5.797648	-0.055616	-0.210383
5	6	0	5.580031	-1.448449	-0.044435
6	6	0	4.311966	-1.979717	0.085196
7	6	0	1.229834	-0.114021	0.035683
8	6	0	2.138326	0.913172	-0.144154
9	7	0	1.892139	-1.317907	0.168393
10	6	0	1.663614	2.332340	-0.192856
11	6	0	0.231646	2.367159	-0.725520
12	6	0	-0.163906	0.142562	0.206073
13	6	0	-2.053761	1.547951	0.175504
14	6	0	-1.227383	-0.813634	0.633468
15	6	0	-2.877354	0.567146	-0.699902
16	6	0	-2.197602	-0.831647	-0.605450
17	7	0	-0.616565	1.335331	-0.096800
18	6	0	-4.365346	0.613549	-0.309928
19	6	0	-4.483921	-1.845827	-0.193010
20	6	0	-5.122783	-0.611747	-0.810060
21	7	0	-3.110816	-1.956879	-0.674207
22	6	0	-1.945618	-0.257523	1.886732
23	6	0	-2.216475	1.245181	1.678140

24	1	0	4.907378	1.893938	-0.385514
25	1	0	6.814159	0.310572	-0.306842
26	1	0	6.436892	-2.113972	-0.018464
27	1	0	4.150928	-3.044663	0.213113
28	1	0	1.467819	-2.231518	0.241704
29	1	0	1.714905	2.772356	0.810988
30	1	0	2.287408	2.944722	-0.848139
31	1	0	-0.236200	3.333385	-0.539927
32	1	0	0.218625	2.170678	-1.804408
33	1	0	-2.290434	2.585682	-0.061756
34	1	0	-0.817845	-1.809599	0.812097
35	1	0	-2.801939	0.899819	-1.740182
36	1	0	-1.539649	-0.924603	-1.477945
37	1	0	-4.797351	1.541749	-0.697803
38	1	0	-4.486226	0.639337	0.777632
39	1	0	-5.007195	-2.754196	-0.504961
40	1	0	-4.571074	-1.780539	0.904992
41	1	0	-6.174654	-0.545405	-0.517240
42	1	0	-5.078044	-0.684383	-1.903354
43	1	0	-2.662568	-2.817045	-0.379738
44	1	0	-2.874840	-0.820148	2.010179
45	1	0	-1.339254	-0.430022	2.777658
46	1	0	-3.206131	1.538232	2.028247
47	1	0	-1.490390	1.854002	2.223165

Nitramidine_conformationB_R.log
Energy: -939.972369 hartree

Center Number	Atomic Number	Atomic Type	Coordinates (Angstroms) X	Y	Z
1	6	0	3.465799	-0.929917	0.047824
2	6	0	3.416780	0.456975	-0.255473
3	6	0	4.578392	1.100053	-0.719261
4	6	0	5.738992	0.357032	-0.870724
5	6	0	5.764780	-1.022191	-0.568049
6	6	0	4.635469	-1.683838	-0.107794
7	6	0	1.382877	-0.213205	0.449799
8	6	0	2.073425	0.885898	0.005166
9	7	0	2.217437	-1.311444	0.481370
10	6	0	1.402135	2.217407	-0.113191
11	6	0	-0.092164	1.976763	-0.321016

12	6	0	-0.011631	-0.186080	0.890252
13	6	0	-2.069549	1.118554	0.913421
14	6	0	-0.626307	-1.234671	1.484604
15	6	0	-2.903505	0.709844	-0.351615
16	6	0	-2.412182	-0.526962	-1.015828
17	7	0	-0.635889	1.054349	0.681701
18	6	0	-4.417657	0.681264	-0.124536
19	6	0	-4.667954	-1.341576	-1.554963
20	6	0	-5.124710	0.092954	-1.344583
21	7	0	-3.191941	-1.408859	-1.530361
22	6	0	-2.026342	-1.143902	2.029472
23	6	0	-2.413378	0.325816	2.176323
24	1	0	4.563678	2.160625	-0.954069
25	1	0	6.644240	0.838268	-1.226943
26	1	0	6.688762	-1.576937	-0.697455
27	1	0	4.657036	-2.743823	0.124929
28	1	0	1.953276	-2.252607	0.730802
29	1	0	1.561382	2.820063	0.789899
30	1	0	1.794982	2.787718	-0.960776
31	1	0	-0.648289	2.913905	-0.229354
32	1	0	-0.244371	1.594629	-1.343578
33	1	0	-2.323419	2.170003	1.087780
34	1	0	-0.065224	-2.149072	1.644262
35	1	0	-2.676468	1.488163	-1.100828
36	1	0	-1.345868	-0.701309	-1.131999
37	1	0	-4.767692	1.695017	0.085413
38	1	0	-4.657956	0.065590	0.748410
39	1	0	-4.980206	-1.741857	-2.519874
40	1	0	-5.035154	-2.006565	-0.767723
41	1	0	-6.207499	0.093779	-1.205109
42	1	0	-4.904003	0.684970	-2.239277
43	1	0	-2.763195	-2.214895	-1.981078
44	1	0	-2.746316	-1.680639	1.391736
45	1	0	-2.079365	-1.639728	3.004124
46	1	0	-3.468580	0.445038	2.428650
47	1	0	-1.836606	0.770033	2.994124

Nitramidine_conformationB_TS.log

Energy: -939.959734 hartree

Center	Atomic	Atomic	Coordinates (Angstroms)		
Number	Number	Type	X	Y	Z

1	6	0	3.196954	-0.983987	0.283814
2	6	0	3.336947	0.329470	-0.240300
3	6	0	4.596230	0.758282	-0.703220
4	6	0	5.662530	-0.120683	-0.638080
5	6	0	5.500230	-1.427087	-0.119396
6	6	0	4.275788	-1.877899	0.344410
7	6	0	1.200532	0.009583	0.393659
8	6	0	2.045502	0.938875	-0.165555
9	7	0	1.891561	-1.151089	0.674822
10	6	0	1.553984	2.314760	-0.486018
11	6	0	0.055792	2.247564	-0.772318
12	6	0	-0.180979	0.296803	0.728633
13	6	0	-2.119200	1.554509	0.271819
14	6	0	-1.048206	-0.606309	1.326860
15	6	0	-2.710894	0.503030	-0.732360
16	6	0	-2.004875	-0.836800	-0.672520
17	7	0	-0.666965	1.477674	0.251867
18	6	0	-4.243273	0.403986	-0.620712
19	6	0	-4.162687	-2.070887	-0.624861
20	6	0	-4.781949	-0.854088	-1.295109
21	7	0	-2.701824	-1.967217	-0.709689
22	6	0	-2.285079	-0.153837	2.068609
23	6	0	-2.580496	1.304627	1.713106
24	1	0	4.723592	1.759501	-1.104154
25	1	0	6.640709	0.190643	-0.989730
26	1	0	6.357551	-2.091721	-0.083374
27	1	0	4.154469	-2.879671	0.743137
28	1	0	1.505014	-1.998913	1.062619
29	1	0	1.746941	2.991556	0.355991
30	1	0	2.061280	2.730442	-1.360821
31	1	0	-0.377558	3.249237	-0.790115
32	1	0	-0.108098	1.785603	-1.757294
33	1	0	-2.414861	2.551337	-0.062400
34	1	0	-0.645750	-1.580209	1.591279
35	1	0	-2.468873	0.883969	-1.734265
36	1	0	-1.031077	-0.904510	-1.145893
37	1	0	-4.677197	1.305791	-1.061995
38	1	0	-4.555955	0.380572	0.426937
39	1	0	-4.440893	-2.996892	-1.129527
40	1	0	-4.484166	-2.146803	0.419880
41	1	0	-5.869198	-0.901087	-1.198804
42	1	0	-4.536128	-0.861586	-2.363143
43	1	0	-2.186252	-2.823350	-0.869039
44	1	0	-3.127682	-0.811708	1.822988

45	1	0	-2.125041	-0.258739	3.146556
46	1	0	-3.634544	1.554352	1.842696
47	1	0	-2.006791	1.977133	2.356709

----

3. REFERENCES

1. Gaussian 16, Revision B.01, Frisch, M. J.; Trucks, G. W.; Schlegel, H. B.; Scuseria, G. E.; Robb, M. A.; Cheeseman, J. R.; Scalmani, G.; Barone, V.; Petersson, G. A.; Nakatsuji, H.; Li, X.; Caricato, M.; Marenich, A. V.; Bloino, J.; Janesko, B. G.; Gomperts, R.; Mennucci, B.; Hratchian, H. P.; Ortiz, J. V.; Izmaylov, A. F.; Sonnenberg, J. L.; Williams-Young, D.; Ding, F.; Lipparini, F.; Egidi, F.; Goings, J.; Peng, B.; Petrone, A.; Henderson, T.; Ranasinghe, D.; Zakrzewski, V. G.; Gao, J.; Rega, N.; Zheng, G.; Liang, W.; Hada, M.; Ehara, M.; Toyota, K.; Fukuda, R.; Hasegawa, J.; Ishida, M.; Nakajima, T.; Honda, Y.; Kitao, O.; Nakai, H.; Vreven, T.; Throssell, K.; Montgomery, J. A., Jr.; Peralta, J. E.; Ogliaro, F.; Bearpark, M. J.; Heyd, J. J.; Brothers, E. N.; Kudin, K. N.; Staroverov, V. N.; Keith, T. A.; Kobayashi, R.; Normand, J.; Raghavachari, K.; Rendell, A. P.; Burant, J. C.; Iyengar, S. S.; Tomasi, J.; Cossi, M.; Millam, J. M.; Klene, M.; Adamo, C.; Cammi, R.; Ochterski, J. W.; Martin, R. L.; Morokuma, K.; Farkas, O.; Foresman, J. B.; Fox, D. J. Gaussian, Inc., Wallingford CT, **2016**.
2. Becke, A. D. Density-functional thermochemistry. IV. The role of the exact exchange. *J. Chem. Phys.* **1993**, *98,* 5648-5652. DOI. 10.1063/1.464913
3. Grimme, S.; Antony, J.; Ehrlich, S.; Krieg, H. A Consistent and Accurate Ab Initio Parametrization of Density Functional Dispersion Correction (DFT-D) for the 94 Elements H-Pu. *J. Chem. Phys.* **2010**, *132*, 154104.
4. Boese, A. D.; Martin, J. M. L. Development of density functionals for thermochemical kinetics. *J. Chem. Phys.* **2004**, *121*, 3405-3416. DOI. 10.1063/1.1774975
5. Yanai, T.; Tew, D. P.; Handy, N. C. A New Hybrid Exchange-Correlation Functional Using the Coulomb-Attenuating Method (CAM-B3LYP). *Chem. Phys. Lett.* **2004**, *393*, 51-57. DOI. 10.1016/j.cplett.2004.06.011
6. Zhao, Y.; Truhlar, D. G. The M06 suite of density functionals for main group thermochemistry, thermochemical kinetics, noncovalent interactions, excited states, and transition elements: two new functionals and systematic testing of four M06-class functionals and 12 other functionals. *Theor. Chem. Account.* **2007**, *120*, 215-241. DOI. 10.1007/s00214-007-0310-x
7. Perdew, P. J.; Burke, K.; Ernzerhof, M. Generalized Gradient Approximation Made Simple. *Phys. Rev. Lett.* **1996**, *77*, 3865-3868. DOI. 10.1103/PhysRevLett.77.3865
8. Adamo, C.; Barone, V. Toward chemical accuracy in the computation of NMR shieldings: the PBE0 model. *Chem. Phys. Lett.* **1999**, *298*, 113-119. DOI. 10.1016/S0009-2614(98)01201-9
9. Tao, J.; Perdew, J. P.; Staroverov, V. N.; Scuseria, G. E. Climbing the Density Functional Ladder: Nonempirical Meta-Generalized Gradient Approximation Designed for Molecules and Solids. *Phys. Rev. Lett.* **2003**, *91*, 146401-4. DOI. 10.1103/PhysRevLett.91.146401
10. Adamo, C.; Barone, V. Exchange functionals with improved long-range behavior and adiabatic connection methods without adjustable parameters: The mPW and mPW1PW models. *J. Chem. Phys.* **1997**, *108*, 664-675. DOI. 10.1063/1.475428
11. Chai, J.-D.; Head-Gordon, M. Systematic optimization of long-range corrected hybrid density functionals. *J. Chem. Phys.* **2008**, *128*, 084106-16. DOI. 10.1063/1.2834918
12. Chai, J.-D.; Head-Gordon, M. Long-range corrected hybrid density functionals with damped atom-atom dispersion corrections. *Phys. Chem. Chem. Phys.* **2008**, *10*,

6615-6620. DOI: 10.1039/b810189b
13. Abraham, M. J.; Murtola, T.; Schulz, R.; Pa´ll, S.; Smith, J. C.; Hess, B.; Lindahl, E. GROMACS: High performance molecular simulations through multi-level parallelism from laptops to supercomputers. *SoftwareX* **2015**, *1-2*, 19-25. DOI. 10.1016/j.softx.2015.06.001
14. Fukui, K. The path of chemical reactions - the IRC approach. *Acc. Chem. Res.* **1981**, *14*, 363-368. DOI. 10.1021/ar00072a001
15. Ishida, K.; Morokuma, K.; Komornicki, A. The intrinsic reaction coordinate. An ab initio calculation for $HNC \rightarrow HCN$ and $H^{-} + CH_4 \rightarrow CH_4 + H^{-}$. *J. Chem. Phys.* **1977**, *66*, 2153-2156. DOI. 10.1063/1.434152
16. Gonzalez, C.; Schlegel, H. B. An improved algorithm for reaction path following. *J. Chem. Phys.* **1989**, *90*, 2154-2161. DOI. 10.1063/1.456010
17. Schlegel, H. B.; Gonzalez, C. Reaction path following in mass-weighted internal coordinates. *J. Phys. Chem.* **1990**, *94*, 5523-5527. DOI. 10.1021/j100377a021
18. Page, M.; Doubleday C.; McIver, J. W. Following steepest descent reaction paths. The use of higher energy derivatives with ab initio electronic structure methods. *J. Chem. Phys.* **1990**, *93*, 5634-5642. DOI. 10.1063/1.459634
19. Cossi, M.; Barone, V.; Mennuci, B.; Tomasi, J. Ab initio study of ionic solutions by a polarizable continuum dielectric model. *Chem. Phys. Lett.* **1998**, *286*, 253-260. DOI. 10.1016/S0009-2614(98)00106-7
20. Tomasi, J.; Mennucci, B.; Cance`s, E. The IEF version of the PCM solvation method: an overview of a new method addressed to study molecular solutes at the QM ab initio level. *J. Mol. Struct. (THEOCHEM)* **1999**, *464*, 211-226. DOI. 10.1016/S0166-1280(98)00553-3
21. Tomasi, J.; Menucci, B.; Cammi, R. Quantum Mechanical Continuum Solvation Models. *Chem. Rev.* **2005**, *105*, 2999-3093. DOI. 10.1021/cr9904009
